# Regulator of calcineurin 1 deletion attenuates mitochondrial dysfunction and apoptosis in acute kidney injury through JNK/Mff signaling pathway

**DOI:** 10.1038/s41419-022-05220-x

**Published:** 2022-09-07

**Authors:** Jing-Jie Xiao, Qing Liu, Ying Li, Fang-Fang Peng, Shan Wang, Zhihan Zhang, Hui Liu, Hong Yu, Shengxiang Tao, Bai-Fang Zhang

**Affiliations:** 1 Department of Biochemistry and Hubei Provincial Key Laboratory of Developmentally Originated Disease, Wuhan University TaiKang Medical School (School of Basic Medical Sciences), Wuhan, Hubei 430071 China; 2 Department of Orthopaedic Trauma and Microsurgery, Zhongnan Hospital of Wuhan University, Wuhan, Hubei 430071 China

**Keywords:** Pathogenesis, Medical research

## Abstract

Ischemia-reperfusion (I/R) induced acute kidney injury (AKI), characterized by excessive mitochondrial damage and cell apoptosis, remains a clinical challenge. Recent studies suggest that regulator of calcineurin 1 (RCAN1) regulates mitochondrial function in different cell types, but the underlying mechanisms require further investigation. Herein, we aim to explore whether RCAN1 involves in mitochondrial dysfunction in AKI and the exact mechanism. In present study, AKI was induced by I/R and cisplatin in RCAN1^*flox/flox*^ mice and mice with renal tubular epithelial cells (TECs)-specific deletion of RCAN1. The role of RCAN1 in hypoxia-reoxygenation (HR) and cisplatin-induced injury in human renal proximal tubule epithelial cell line HK-2 was also examined by overexpression and knockdown of RCAN1. Mitochondrial function was assessed by transmission electron microscopy, JC-1 staining, MitoSOX staining, ATP production, mitochondrial fission and mitophagy. Apoptosis was detected by TUNEL assay, Annexin V-FITC staining and Western blotting analysis of apoptosis-related proteins. It was found that protein expression of RCAN1 was markedly upregulated in I/R- or cisplatin-induced AKI mouse models, as well as in HR models in HK-2 cells. RCAN1 deficiency significantly reduced kidney damage, mitochondrial dysfunction, and cell apoptosis, whereas RCAN1 overexpression led to the opposite phenotypes. Our in-depth mechanistic exploration demonstrated that RCAN1 increases the phosphorylation of mitochondrial fission factor (Mff) by binding to downstream c-Jun N-terminal kinase (JNK), then promotes dynamin related protein 1 (Drp1) migration to mitochondria, ultimately leads to excessive mitochondrial fission of renal TECs. In conclusion, our study suggests that RCAN1 could induce mitochondrial dysfunction and apoptosis by activating the downstream JNK/Mff signaling pathway. RCAN1 may be a potential therapeutic target for conferring protection against I/R- or cisplatin-AKI.

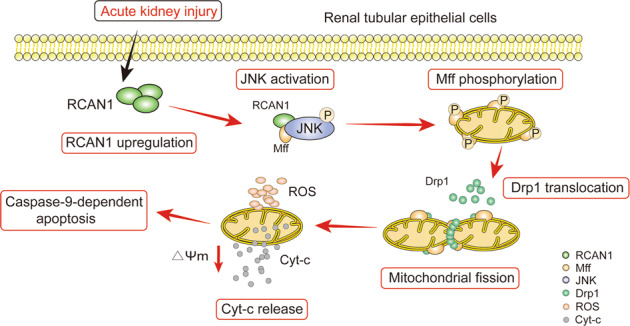

## Introduction

Acute kidney injury (AKI) is a common disease worldwide characterized by a dramatic decline in renal function, occurring in up to 20% of hospitalized patients and up to 17.5% of cancer patients. Common causes of AKI include ischemia-reperfusion (I/R) injury, sepsis, and exposure to nephrotoxic substances such as cisplatin and contrast agents [1, 2]. AKI disrupts the cellular redox balance and induces excessive production of ROS in the kidney, leading to a series of events including loss of tubular function, mitochondrial damage, energy depletion, and cell death of renal tubular epithelial cells (TECs) [3]. Despite significant progress in the pathogenesis of AKI over the past few decades, there remains a lack of effective intervention.

Regulator of calcineurin 1 (RCAN1) belongs to a family of endogenous regulators of calcineurin. *Rcan1* gene consists of seven exons and six introns, which can generate different isoforms through alternative splicing and express different alternatively spliced proteins [4, 5]. Recent studies have shown that RCAN1 plays a direct role in the regulation of mitochondrial function. RCAN1 produces a more confluent mitochondrial network, enhances mitochondrial function, and reduces apoptosis in cardiomyocytes, but RCAN1 overexpression causes mitochondrial dysfunction and induces apoptosis in neuronal cells and β cells [6–9]. These opposing results suggest that RCAN1 functions related to mitochondrial regulation appear to be dependent on different cellular contexts. Mitochondrial dysfunction is often observed in TECs when AKI occurs [10]. However, the role of RCAN1 in regulating mitochondrial function in tubular cells remains poorly defined. Additionally, whether RCAN1 plays a crucial role in mitochondrial regulation in AKI remains unknown.

In the present study, we showed that RCAN1 accelerated the progression of AKI by inducing mitochondrial fragmentation and apoptosis. Mechanistically, RCAN1 activated the downstream c-Jun N-terminal kinase (JNK)/mitochondrial fission factor (Mff) signaling pathway, and RCAN1 directly interacted with JNK and Mff. In conclusion, our findings reveal a damaging effect of RCAN1 in AKI and suggest that RCAN1 might be a novel target for the treatment and prevention of AKI.

## Results

### Deletion of tubule RCAN1 reduces renal dysfunction, mitochondrial damage, and apoptosis in AKI mice

**Fig. 1:**
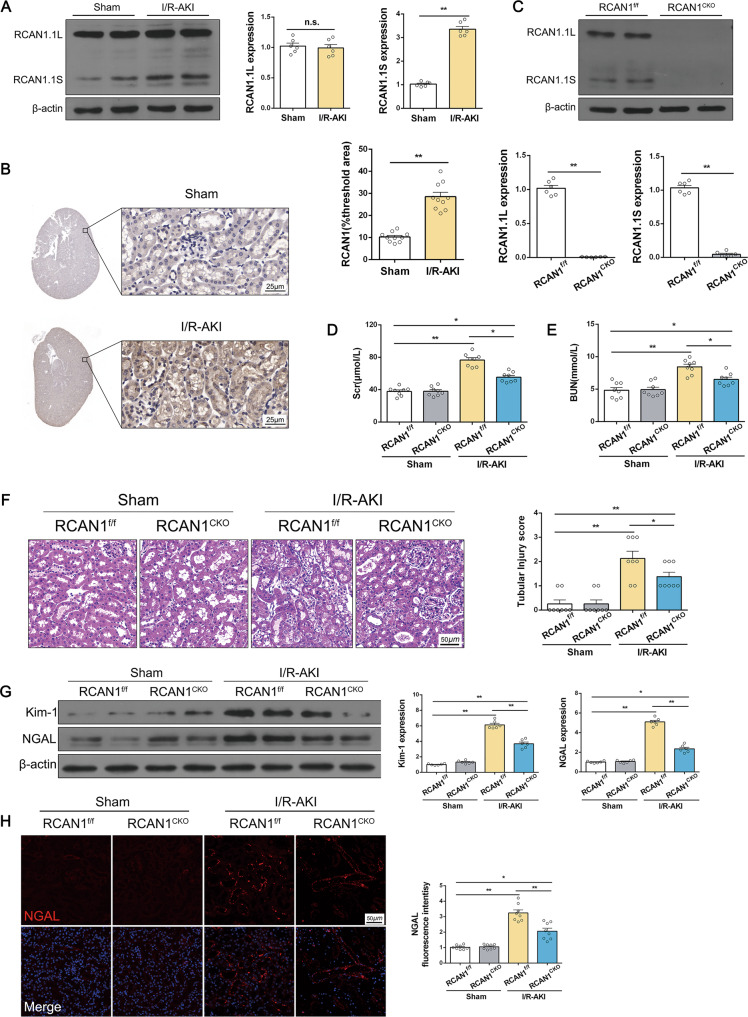
RCAN1 deletion attenuated I/R-initiated renal damage. **A**, **B** WT mice were subjected to I/R injury. The kidneys of sham operation or I/R-AKI were isolated, and RCAN1 was monitored via Western blotting and IHC. Scale bar, 25 μm. *n* = 6. **C** RCAN1 expression in RCAN1^f/f^ and RCAN1^CKO^ mice was detected by Western blotting (*n* = 6). **D**, **E** Scr and BUN were measured using an assay kit (*n* = 8). **F** HE staining was conducted to observe I/R-mediated renal damage. Scale bar, 50 μm (*n* = 6). **G** The protein levels of Kim-1 and NGAL in kidney tubular injury were evaluated by Western blotting (*n* = 6). **H** An immunofluorescence (IF) assay was performed to analyze the expression of NGAL in response to renal I/R injury. Scale bar, 50 μm. *n* = 3 *p < 0.05, **p < 0.01.

Renal I/R injury in wild type mice was induced in vivo by applying 30 min of ischemia followed by 24 h of reperfusion. Compared with the sham group, RCAN1.1 S expression increased in the reperfused kidneys, but RCAN1.1 L expression was not significantly changed (Fig. 1A). We next induced mouse AKI by cisplatin injection and similar results were observed (Fig. S2A). Immunohistochemistry staining also showed RCAN1 upregulation compared with the sham group (Fig. 1B). To investigate the role of RCAN1 in renal I/R injury, RCAN1-conditional knockout (RCAN1^CKO^) mice were constructed (Fig. S1, Fig. 1C.). Compared with the sham group, the serum creatinine and urea nitrogen significantly increased in the mice treated with I/R and cisplatin injury and were downregulated in RCAN1^CKO^ mice (Fig. 1D, E, Fig. S2B, C). Next, alterations in renal histology were observed. As shown in Fig. 1F and Fig. S2D, compared with the sham group, AKI-induced proximal tubular damage, as evidenced via HE staining. Interestingly, RCAN1 deletion attenuated tubular damage compared with the kidneys of the model group. Additionally, we examined the expression of Kim-1 and neutrophil gelatinase-associated lipocalin (NGAL), which are indicators of kidney injury [11]. Increased protein levels of Kim-1 and NGAL after I/R injury were prevented in RCAN1^CKO^ mice (Fig. 1G). Furthermore, the immunofluorescence (IF) assay also showed that RCAN1 deletion inhibited the upregulation of NGAL in the kidney of I/R injury mice (Fig. 1H).

**Fig. 2:**
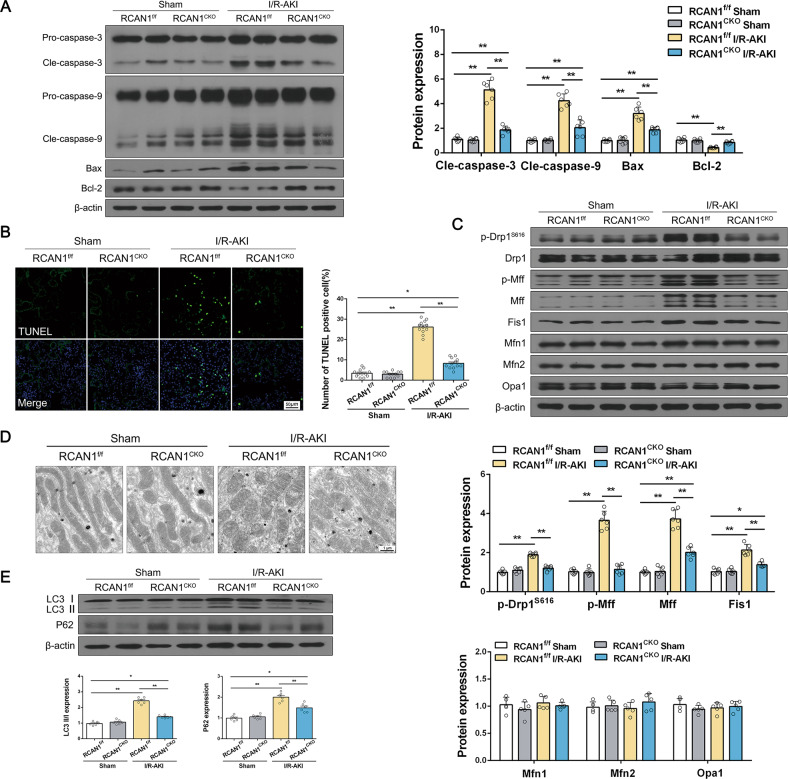
RCAN1 deletion reduced I/R-initiated mitochondrial damage and apoptosis. **A** Western blotting was performed to analyze the expression of Pro-caspase-3, Cle-caspase-3, Pro-caspase-9, Cle-caspase-9, Bax, and Bcl-2 from sham-operated and I/R-AKI kidneys of RCAN1^f/f^ and RCAN1^CKO^ mice (*n* = 6). **B** A TUNEL assay was conducted to observe cell death. The number of TUNEL-positive cells was counted in the right panel. Scale bar, 50 μm. *n* = 3. **C** Western blotting was performed to analyze the expression of p-Drp1^S616^, Drp1, p-Mff, Mff, Fis1, Mfn1, Mfn2, and Opa1 from sham-operated and I/R-AKI kidneys of RCAN1^f/f^ and RCAN1^CKO^ mice (*n* = 6). **D** Representative TEM micrographs of mitochondrial morphology from sham-operated and I/R-AKI kidneys of RCAN1^f/f^ and RCAN1^CKO^ mice. Scale bar, 1 μm. **E** Western blotting was performed to analyze the protein levels of LC3 and P62 (*n* = 6). *p < 0.05, **p < 0.01.

Additionally, as shown in Fig. 2A and Fig. S2E, AKI upregulated the expression of proteins related to cell apoptosis, such as cleaved-caspase-3, cleaved-caspase-9, and Bax, and downregulated the level of anti-apoptotic protein Bcl-2. RCAN1 deletion reversed the balance between anti- and pro-apoptotic factors. This finding was further supported by the results of the TUNEL assay. Compared with the sham group, TUNEL-positive cells increased to about ~27%, whereas RCAN1 deletion repressed the apoptotic index to ~10% (Fig. 2B, Fig. S2F).

We next observed mitochondrial fission, which has been shown to be modulated by Drp1 phosphorylation [12, 13]. As shown in Fig. 2C and Fig. S2G, the protein level of phosphorylated Drp1 at Ser616 was upregulated in renal tissue of I/R and cisplatin-induced AKI mice. However, AKI did not influence the total level of Drp1. Moreover, mitochondrial fission factors such as phospho-Mff (p-Mff), Mff, and Fis1 were upregulated in response to AKI, which were reversed by RCAN1 deletion (Fig. 2C, Fig. S2G). Additionally, we observed mitochondrial fusion factors such as Mfn1, Mfn2, and Opa1, which could be considered antagonists of mitochondrial fission. Unexpectedly, compared with the sham group, AKI had no influence on the total level of these proteins (Fig. 2C, Fig. S2G). Mitochondrial ultrastructural changes were also observed by electron microscopy. As shown in Fig. 2D, no noticeable ultrastructural changes were observed in mitochondria in RCAN1^CKO^ mice. But the percentage of tubular cells with fragmented mitochondria increased in I/R-induced AKI mice, and TECs-specific knockout of RCAN1 attenuated mitochondrial fragmentation.

It has been reported that the activation of mitochondrial fission is related to the induction of mitophagy [14]. Therefore, we determined the expression of the autophagy marker LC3 and found that the levels of LC3-I and LC3-II in the I/R-induced injury model kidneys were significantly higher than those in sham-operated kidneys (Fig. 2E). LC3 may accumulate due to the increased upstream formation of autophagosomes or the impaired fusion of downstream autophagosome-lysosomes. To distinguish between these two possibilities, we examined P62, a selective substrate of autophagy. Also, the expression of P62 remarkably increased in injured kidneys compared with the sham group (Fig. 2E). The increase in both LC3 and P62 expression in injury kidneys demonstrated that LC3 accumulation is likely attributable to the inhibition of autophagosome clearance, suggesting impaired autophagy flux in injury kidneys. In addition, the expression of P62 was downregulated in RCAN1^CKO^ mice, suggesting that blocked autophagy flux was improved in I/R-induced AKI. All of the findings indicate that deletion of tubule RCAN1 reduces renal dysfunction, mitochondrial damage, and apoptosis in I/R and cisplatin-induced AKI mice.

### RCAN1 silencing alleviates HR and cisplatin injury by improving mitochondrial dysfunction and inhibiting apoptosis

**Fig. 3:**
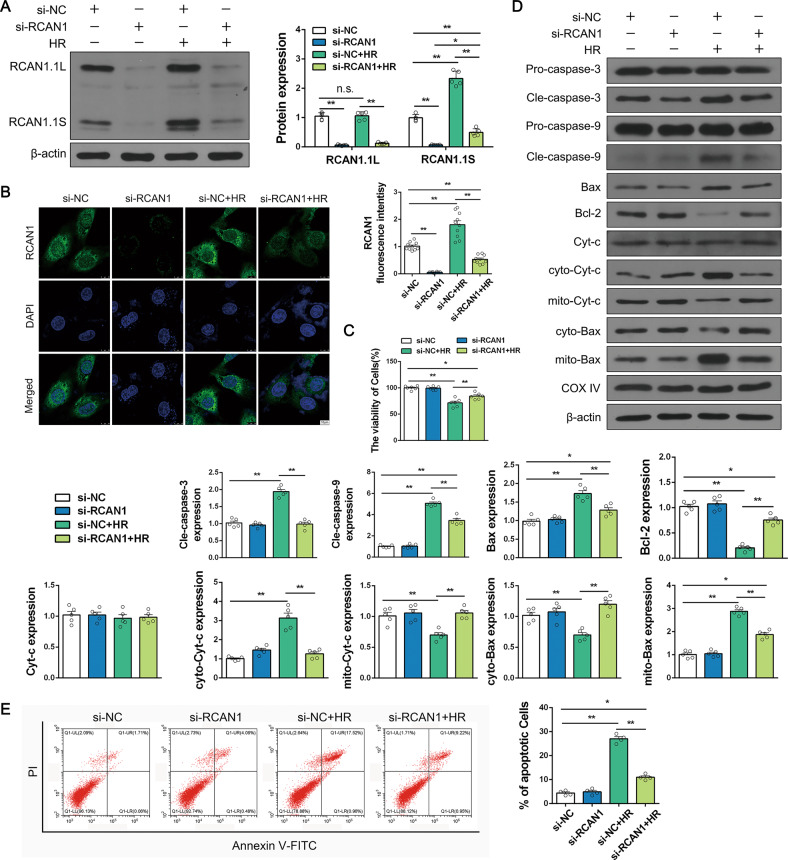
RCAN1 silencing alleviated HR injury by inhibiting apoptosis. **A** Renal TEC line HK-2 was used with a hypoxia and reoxygenation (HR) stimulus to mimic animal I/R injury. siRNA against RCAN1 and si-NC were transfected into HK-2 cells. The alteration of RCAN1 was detected by Western blotting (*n* = 5). **B** An IF assay for RCAN1 and DAPI was used to tag the nucleus. Scale bar, 10 μm. *n* = 3. **C** A CCK-8 assay was performed to analyze cellular viability (*n* = 5). **D** The proteins of cytoplasm and mitochondria were fractionated, and Western blotting was performed to analyze the expression of Pro-caspase-3, Cle-caspase-3, Pro-caspase-9, Cle-caspase-9, Bax, Bcl-2, Cyt-c, cyto-Cyt-c, mito-Cyt-c, cyto-Bax, and mito-Bax, with β-actin as the loading control for cytoplasm and COX IV for mitochondria. *n* = 5. **E** HK-2 cells were stained with Annexin V-FITC and PI to determine cell apoptosis using a flow cytometry assay (*n* = 4). *p < 0.05, **p < 0.01.

Subsequently, HK-2 cells were used in vitro with a hypoxia/reoxygenation (HR) and cisplatin stimulus to mimic animal AKI. To further provide more solid evidence for the role of RCAN1 in AKI, siRNA against RCAN1 was transfected into the HK-2 cell line (Fig. 3A, B, Fig. S5A). Compared with the control group, HR and cisplatin significantly increased the expression of RCAN1.1 S but had no significant effect on RCAN1.1 L, which was consistent with the results in AKI kidney tissues. In addition, IF also showed that the expression of RCAN1 was upregulated in the HR group, and RCAN1 was mainly distributed in the cytoplasm (Fig. 3B). Additionally, HR injury significantly reduced the cell viability of HK-2 cells, and HR-mediated cell death was mostly repressed by RCAN1 silencing (Fig. 3C). As shown in Fig. 3D and Fig. S5B, HR and cisplatin injury upregulated the expression of cle-caspase-3, cle-caspase-9, and Bax, while the protein level of Bcl-2 was downregulated in the injured tissue. More importantly, RCAN1 silencing could reverse the balance between anti- and pro-apoptotic factors. Next, we fractionated proteins of cytoplasm and mitochondria, and then detected the expression levels of Cytochrome-c (Cyt-c) and Bax. Compared with the control group, HR promoted Bax migration to mitochondria and therefore reduced the levels of cytoplasmic Bax. At the same time, the pro-apoptotic factor Cyt-c was released from the mitochondria into the cytoplasm after HR injury. Silencing of RCAN1 limited Bax translocation and Cyt-c leakage (Fig. 3D). Moreover, Annexin V-FITC/PI staining showed that RCAN1 knockdown reduced HR-induced apoptosis in HK-2 cells (Fig. 3E).

**Fig. 4:**
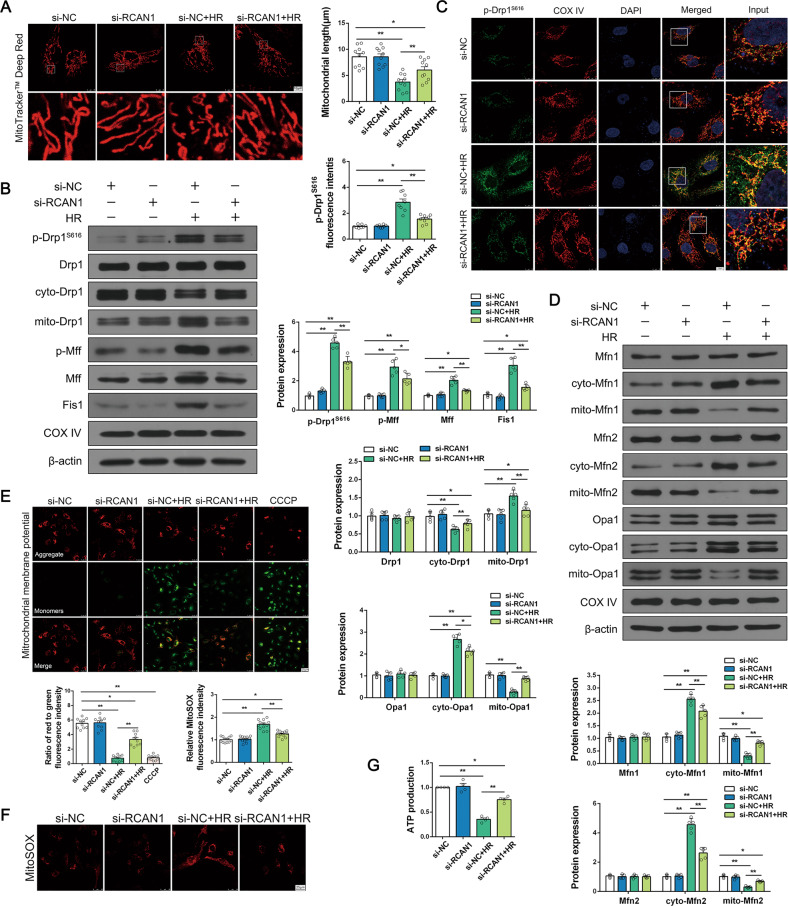
RCAN1 silencing attenuated HR injury by regulating mitochondrial dysfunction. **A** Mitochondrial morphology of HK-2 was assessed by MitoTracker^TM^ Deep Red staining, and the average length of mitochondria was measured. Scale bar, 10 μm. *n* = 3. **B** The proteins of cytoplasm and mitochondria were fractionated, and Western blotting was performed to analyze the expression of p-Drp1^S616^, Drp1, cyto-Drp1, mito-Drp1, p-Mff, Mff, and Fis1, with β-actin used as the loading control for cytoplasm and COX IV for mitochondria. *n* = 5. **C** The co-localization of p-Drp1^S616^ and mitochondria was detected by IF, and the mitochondria were labeled with the COX IV antibody. Scale bar, 10 μm. *n* = 3. **D** The proteins of cytoplasm and mitochondria were fractionated, and Western blotting was performed to analyze the expression of Mfn1, cyto-Mfn1, mito-Mfn1, Mfn2, cyto-Mfn2, mito-Mfn2, Opa1, cyto-Opa1, and mito-Opa1, with β-actin as the loading control for cytoplasm and COX IV for mitochondria. *n* = 5. **E** The mitochondrial potential was observed via JC-1 staining. The red to green fluorescence ratio was recorded to quantify the mitochondrial potential (rate). Scale bar, 25 μm. *n* = 3. **F** Mitochondrial ROS levels were detected by MitoSOX and analyzed by confocal microscopy. Scale bar, 25 μm. *n* = 3. **G** ATP production was measured to reflect mitochondrial function. *n* = 4. *p < 0.05, **p < 0.01.

To further explain the effects of RCAN1 on HR injury, we focused on mitochondrial fission. Drp1-related mitochondrial fission is noted as a critical step in aggravating HR injury [15]. As shown in Fig. 4A and Fig. S5C, the mitochondrial morphology changed from an elongated network into small spheres or short rods after HR and cisplatin injury. Interestingly, the silencing of RCAN1 reduced mitochondrial fragmentation. Next, we measured the protein levels of mitochondrial fission via Western blotting. Compared with the control group, the protein levels of phosphorylated Drp1 at Ser616 but not total Drp1 was upregulated in the HR group and cisplatin group. Similarly, mitochondrial fission factors p-Mff, Mff, and Fis1 were upregulated, and these effects were reversed by RCAN1 silencing (Fig. 4B, Fig. S5D). Given that Drp1 translocation onto the surface of mitochondria is the prerequisite for mitochondrial fission, we fractionated proteins of the cytoplasm and mitochondria, and detected the expression levels of Drp1. Compared to the control group, HR promoted Drp1 migration to mitochondria and reduced cytoplasmic Drp1. In the meantime, IF was used to observe the co-location of p-Drp1^S616^ and mitochondria. As shown in Fig. 4C, loss of RCAN1 maintained the mitochondrial network and blocked Drp1 translocation to mitochondria. Although the total levels of mitochondrial fusion factors Mfn1, Mfn2, and Opa1 did not change, HR injury reduced the content of these proteins in mitochondria, and knockdown of RCAN1 blocked their reduction (Fig. 4D).

Accordingly, mitochondrial homeostasis was measured with or without RCAN1 silencing in the setting of HR and cisplatin injury. Firstly, to explain the role of fission in cellular damage, we focused on the mitochondrial membrane potential and mito-ROS. Mitochondrial membrane potential, assessed by JC-1 staining, was decreased under HR and cisplatin injury and was increased to near-normal levels with RCAN1 silencing (Fig. 4E, Fig. S5E). Notably, as shown in Fig. 4F and Fig. S5F, HR and cisplatin injury drove HK-2 cells to produce excessive ROS as demonstrated via MitoSOX staining, but RCAN1 knockdown reduced ROS level. Mitochondrial function was then monitored via ATP production. Compared with the control group, HR injury suppressed the concentration of cellular ATP, and this effect was nullified by RCAN1 silencing (Fig. 4G).

In addition to mitochondrial fission, mitophagy is another mechanism that preserves mitochondrial homeostasis. Fission is reportedly accompanied by mitophagy, which aggravates mitochondrial injury facilitating cellular death via excessive self-consumption [16]. As shown in Fig. S4A, B and Fig. S5G, we noticed that HR and cisplatin injury robustly increased mitophagy markers, including LC3, P62, PINK1, Parkin, and BNIP3. However, these increases were partly prevented by the knockdown of RCAN1. Furthermore, we used the IF staining of COX IV, P62, and BNIP3 to observe mitophagy. Our results clearly showed that HR injury promoted the co-localization of P62 and BNIP3 with mitochondria, suggesting impaired mitophagy flux in injury HK-2. RCAN1 silencing reduced P62 and BNIP3 co-localization (Fig. S4C, D), suggesting that impaired mitophagy flux was improved. These data collectively indicate that silencing RCAN1 under HR injury improved mitochondrial dysfunction and reduced mitochondrial-dependent cellular apoptosis. Taken together, these data illustrate that RCAN1 plays a crucial role in HR and cisplatin-induced mitochondrial dysfunction and apoptosis.

### JNK/Mff signaling is involved in RCAN1-mediated HR injury

**Fig. 5:**
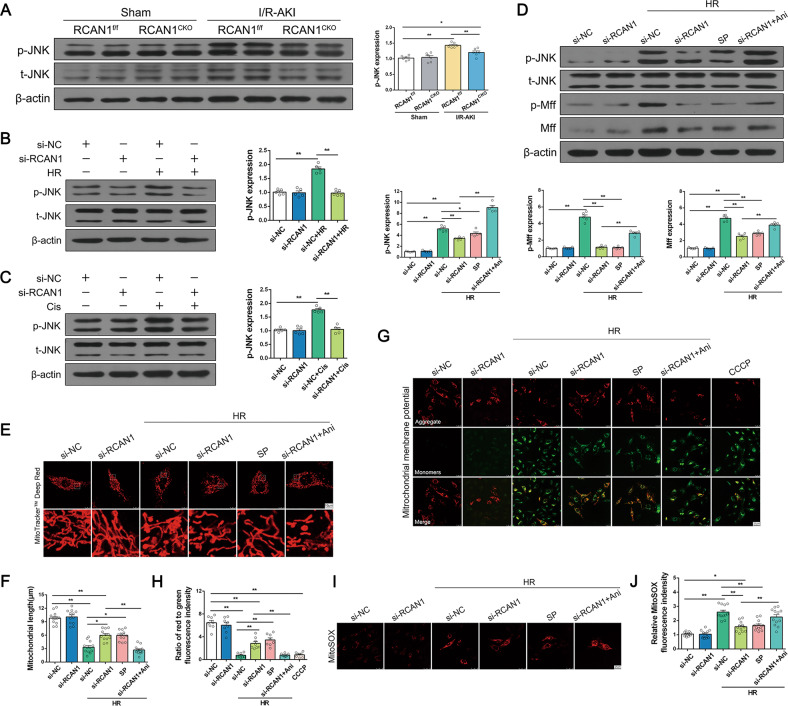
RCAN1-induced mitochondrial dysfunction via the JNK/Mff pathway. **A** Western blotting was performed to analyze the expression of p-JNK and JNK from sham-operated and I/R-AKI kidneys of RCAN1^f/f^ and RCAN1^CKO^ mice (*n* = 6). **B**, **C** HK-2 cells were treated with HR or cisplatin, and the expression levels of p-JNK and JNK were detected by Western blotting (*n* = 5). **D** HK-2 cells were treated with JNK inhibitor SP600125 (SP) and JNK activator Anisomycin (Ani) and then exposed to HR. Western blotting was used to evaluate changes in p-JNK, JNK, p-Mff, and Mff expression (*n* = 5). **E**, **F** HK-2 cells were treated with SP and Ani, and mitochondrial morphology of HK-2 was assessed by MitoTracker^TM^ Deep Red staining, and the average length of mitochondria was measured. Scale bar, 10 μm. *n* = 3. **G**, **H** HK-2 cells were treated with SP and Ani, and the mitochondrial potential was observed via JC-1 staining. The ratio of red to green fluorescence was recorded to quantify the mitochondrial potential (rate). *n* = 3. **I**, **J** Mitochondrial ROS levels were detected by MitoSOX and analyzed by confocal microscopy (*n* = 3). Scale bar, 25 μm. *p < 0.05, **p < 0.01.

Given that the change of Mff and its phosphorylation was the most significant in the proteins related to mitochondrial division after I/R and RCAN1 deletion, we next focused on Mff. The JNK pathway has been found to be the upstream activator for Mff in the cardiac I/R model [17], suggesting that the regulatory effect of RCAN1 on Mff may rely on JNK activity. As shown in Fig. 5A, compared with the sham group, JNK phosphorylation (p-JNK) level significantly increased in the mice treated with I/R injury and was downregulated in RCAN1^CKO^ mice. Meanwhile, the protein level of p-JNK was significantly increased in HR injury, as well as cisplatin treatment. Loss of RCAN1 prevented the upregulation of p-JNK (Fig. 5B, C). These results indicate that JNK is involved in HR and cisplatin-induced injury, and RCAN1 may regulate JNK activity. Both the silencing of RCAN1 and the inhibition of JNK via SP600125 (SP) markedly reduced HR-induced upregulation of p-Mff and Mff. In contrast, reactivation of JNK in RCAN1-silenced cells via Anisomycin (Ani) re-elevated the expression of p-Mff and Mff (Fig. 5D). Collectively, these data suggest that RCAN1 under HR injury led to an increase in Mff-related mitochondrial fission that occurred at least partially through JNK-mediated Mff phosphorylation.

To further investigate the effects of JNK on mitochondria, we observed mitochondrial morphology, mitochondrial membrane potential, and mitochondrial ROS by confocal microscopy. As shown in Fig. 5E–J, SP restored partial mitochondrial morphology to normal levels, decreased mitochondrial ROS release, and increased mitochondrial membrane potential under HR injury, which was consistent with RCAN1 silencing. The role of Ani was opposite to SP.

**Fig. 6:**
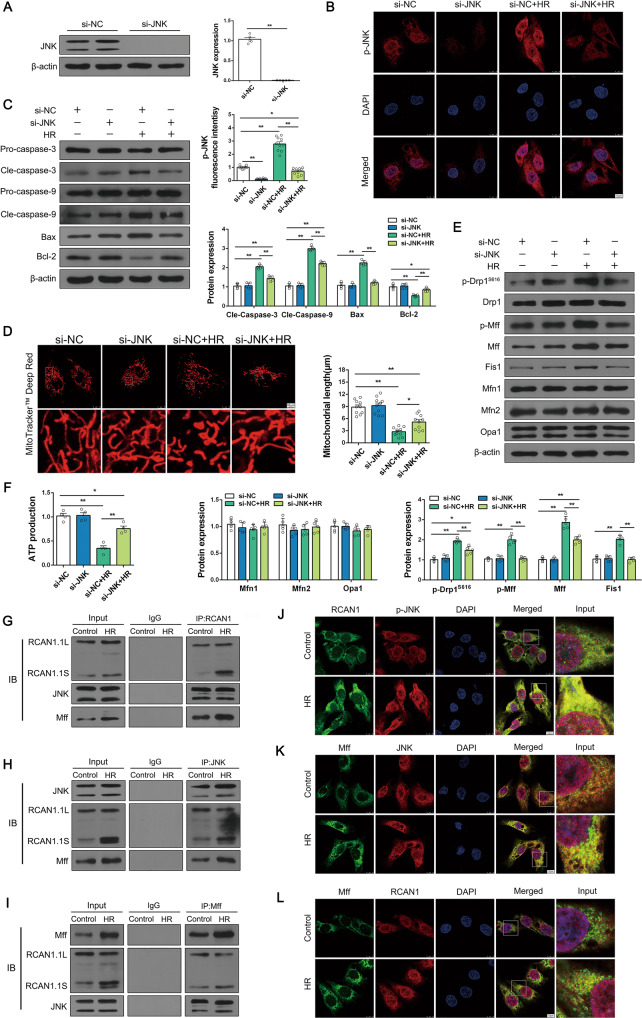
JNK silencing alleviated HR injury by regulating mitochondrial dysfunction and inhibiting apoptosis, and RCAN1, JNK, and Mff all bound together. **A** siRNA against JNK and si-NC were transfected into cells. The expression level of JNK was monitored via Western blotting. (*n* = 5) **B** An IF assay for p-JNK, and DAPI was used to tag the nucleus. Scale bar, 10 μm. *n* = 3. **C** The renal tubular epithelial cell line HK-2 was used with HR, and siRNA against JNK and si-NC were transfected into cells. The expression levels of Pro-caspase-3, Cle-caspase-3, Pro-caspase-9, Cle-caspase-9, Bax, and Bcl-2 were detected by Western blotting (*n* = 5). **D** Mitochondrial morphology of HK-2 was assessed by MitoTracker^TM^ Deep Red staining, and the average length of mitochondria was measured. Scale bar, 10 μm *n* = 3. **E** Western blotting was performed to analyze the expression of p-Drp1^S616^, Drp1, p-Mff, Mff, Fis1, Mfn1, Mfn2, and Opa1 in HK-2 cells (*n* = 5). **F** Mitochondrial function was measured with an ATP assay kit (*n* = 4). **G**–**I** Representative Co-IP analysis of RCAN1, JNK, and Mff in HK-2 cells under HR injury (*n* = 3). **J**–**L** The co-localization of RCAN1 and p-JNK, Mff, and JNK, as well as Mff and RCAN1, were detected by IF, and DAPI was used to tag the nucleus. Scale bar, 10 μm. *n* = 3. *p < 0.05, **p < 0.01.

To further explore the role of JNK, we silenced JNK with siRNA. As shown in Fig. 6A, B, Western blotting and IF staining showed that JNK silencing was effective. In addition, p-JNK was mainly distributed in the cytoplasm and nucleus of HK-2 cells (Fig. 6B). It was evident that JNK silencing attenuated HR injury-induced apoptosis and excessive mitochondrial division (Fig. 6C, E). Meanwhile, mitochondrial morphology staining showed that silencing of JNK reduced HR-induced mitochondrial fragmentation (Fig. 6D). Additionally, mitochondrial function was monitored via ATP production. Compared with the control group, HR injury suppressed the concentration of cellular ATP, and this effect was nullified by JNK silencing (Fig. 6F). Taken together, these data clearly show that the RCAN1-JNK/Mff pathway was the upstream regulator of HR-activated mitochondrial dysfunction and apoptosis.

Given that RCAN1 affected mitochondrial fission and apoptosis via the JNK/Mff signaling pathway, we next explored the relationship between RCAN1, JNK, and Mff. First, we performed Co-immunocoprecipitation (IP) assays between RCAN1, JNK, and Mff, and found a strong interaction between endogenous RCAN1, JNK, and Mff in HK-2 cells under HR injury (Fig. 6G–I). The results of IF and Western blotting showed that RCAN1 resided in the cytoplasm but not mitochondria, and it was clear that RCAN1 and p-JNK were co-localized mainly in the cytoplasm (Fig. 6J, Fig. S3). Confocal microscopy also showed co-localization of JNK with Mff and RCAN1 with Mff (Fig. 6K, L). We found that RCAN1, JNK, and Mff all bound together in HK-2 cells.

### HK-2-specific RCAN1 overexpression aggravates HR injury

**Fig. 7:**
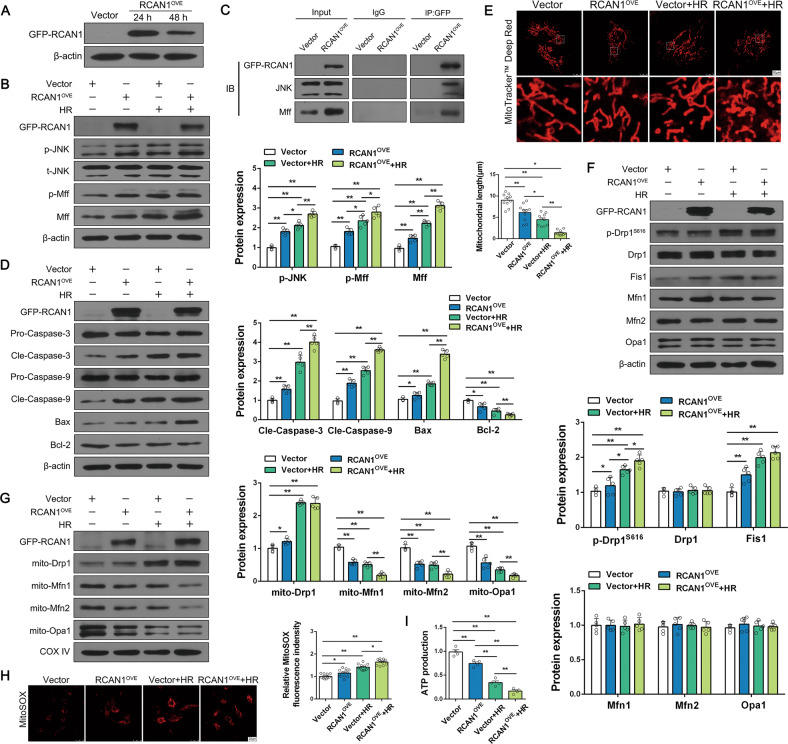
HK-2-specific RCAN1 overexpression aggravated HR injury. **A** HK-2 cells were transfected by empty vector and pCMV-EGFP-RCAN1 (RCAN1^OVE^) for 24 h or 48 h, and the protein level of GFP-RCAN1 was assayed by Western blotting (*n* = 3). **B** HK-2 cells were used with HR and then treated with or without RCAN1^OVE^, and protein levels of GFP-RCAN1, p-JNK, t-JNK, p-Mff, and Mff were assayed by Western blotting (*n* = 5). **C** Representative Co-IP analysis of GFP-RCAN1, JNK, and Mff in HK-2 cells under RCAN1^OVE^ (*n* = 3). **D** The expression levels of GFP-RCAN1, Pro-caspase-3, Cle-caspase-3, Pro-caspase-9, Cle-caspase-9, Bax, and Bcl-2 were detected by Western blotting (*n* = 5). **E** Mitochondrial morphology of HK-2 was assessed by MitoTracker^TM^ Deep Red staining, and the average length of mitochondria was measured. Scale bar, 10 μm. *n* = 3. **F** Western blotting was performed to analyze the expression of GFP-RCAN1, p-Drp1^S616^, Drp1, Fis1, Mfn1, Mfn2, and Opa1 in HK-2 cells (*n* = 5). **G** The proteins of mitochondria were fractionated, and Western blotting was performed to analyze the expression of mito-Drp1, mito-Mfn1, mito-Mfn2, and mito-Opa1, and COX IV was used as the loading control for mitochondria (*n* = 5). **H** Mitochondrial ROS levels were detected by MitoSOX and then analyzed by confocal microscopy. Scale bar, 25 μm. *n* = 3. **I** ATP production was measured to reflect mitochondrial function. *n* = 3. *p < 0.05, **p < 0.01.

To investigate the effects of RCAN1 on the JNK signaling pathway, mitochondrial fission and apoptosis, RCAN1 was overexpressed by transfecting GFP-RCAN1 in HK-2 cells (Fig. 7A). As shown in Fig. 7B and D, RCAN1 overexpression (RCAN1^OVE^) upregulated the protein levels of p-JNK, p-Mff, and proteins related to apoptosis, such as cle-caspase-3, cle-caspase-9, and Bax, and downregulated Bcl-2 with or without HR injury. Co-IP assays in HK-2 cells after RCAN1^OVE^ also showed a constitutive interaction between GFP-RCAN1 with JNK and Mff, similar to the above findings of HR injury (Fig. 7C). As seen in Fig. 7E, confocal microscopy showed that mitochondria changed into small spheres or short rods after RCAN1^OVE^. The expression of p-Drp1^S616^ and Fis1 was also upregulated (Fig. 7F). RCAN1^OVE^ promoted Drp1 migration to mitochondria, and the contents of Mfn1, Mfn2, and Opa1 were decreased in mitochondria under RCAN1^OVE^ (Fig. 7G). Moreover, as shown in Fig. 7H, I, RCAN1^OVE^ further increased the mitochondrial ROS and decreased the ATP production compared to the HR group. Taken together, these results demonstrate that RCAN1^OVE^ aggravated mitochondrial dysfunction and apoptosis during HR injury.

### JNK silencing alleviates RCAN1 overexpression-induced mitochondrial dysfunction and apoptosis

**Fig. 8:**
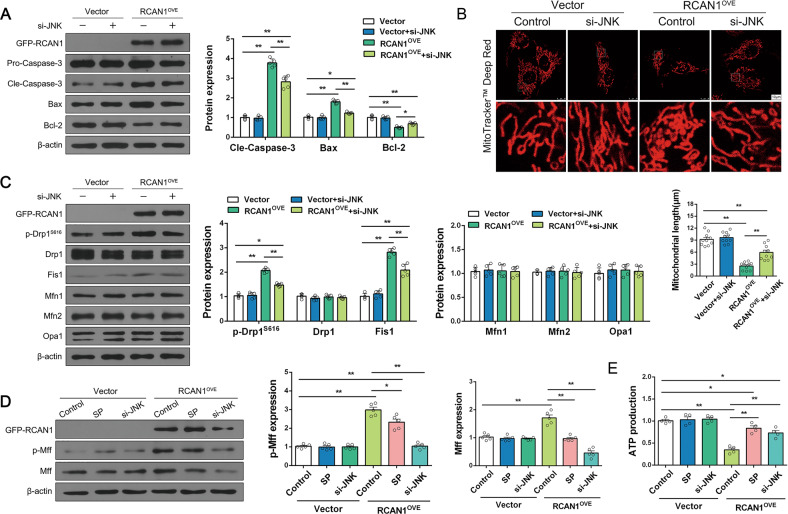
JNK silencing ameliorated RCAN1 overexpression-induced mitochondrial dysfunction and apoptosis. **A** HK-2 cells were transfected by empty vector and RCAN1^OVE^, and siRNA against JNK was transfected into cells. Protein levels of GFP-RCAN1, Pro-caspase-3, Cle-caspase-3, Bax, and Bcl-2 were assayed by Western blotting (*n* = 5). **B** Mitochondrial morphology of HK-2 was assessed by MitoTracker^TM^ Deep Red staining, and the average length of mitochondria was measured. Scale bar, 10 μm. *n* = 3. **C** Western blotting was performed to analyze the expression of GFP-RCAN1, p-Drp1^S616^, Drp1, Fis1, Mfn1, Mfn2, and Opa1 in HK-2 cells (*n* = 5). **D** HK-2 cells were treated with SP or siRNA against JNK under RCAN1^OVE^, and the protein levels of GFP-RCAN1, p-Mff, and Mff in cells were assayed by Western blotting (*n* = 5). **E** ATP production was measured to reflect mitochondrial function. *n* = 3. *p < 0.05, **p < 0.01.

Finally, we evaluated whether JNK activity was required for RCAN1^OVE^-induced mitochondrial fission and apoptosis. We thus overexpressed RCAN1 by transfecting GFP-RCAN1 and silenced JNK with siRNA to inhibit JNK function. Similar to previous results, Western blotting results further validated that overexpressed RCAN1 upregulated the expression of cle-caspase-3 and Bax, downregulated Bcl-2, and JNK silencing reversed the balance between anti- and pro-apoptotic factors (Fig. 8A). Moreover, silencing of JNK reduced mitochondrial fragmentation and upregulation of p-Drp1^S616^ and Fis1 in RCAN1^OVE^ cells (Fig. 8B, C). Furthermore, SP and si-JNK reduced the phosphorylation level of Mff and the total Mff induced by RCAN1^OVE^ (Fig. 8D). Finally, compared with the control group, RCAN1^OVE^ suppressed the concentration of cellular ATP, and this effect was nullified by JNK silencing (Fig. 8E). Taken together, these results suggest that JNK activity is essential for the exacerbating impact of RCAN1^OVE^ on HR injury.

## Discussion

Numerous studies have shown that RCAN1 is involved in pathophysiological processes such as Alzheimer’s disease, myocardial ischemia-reperfusion injury, and diabetes [18–20]. However, the role of RCAN1 in AKI is unclear. In this study, we found the following: (1) the expression of RCAN1 was significantly upregulated in I/R- or cisplatin-induced AKI; (2) RCAN1 knockout alleviated renal injury, mitochondrial dysfunction, and caspase-9-dependent apoptosis in AKI; (3) I/R, HR or cisplatin all led to the increase of JNK phosphorylation and the upregulation of Mff; (4) silencing of JNK alleviated HR-induced mitochondrial dysfunction and apoptosis, and activation of JNK increased the expression of p-Mff and Mff, triggering excessive mitochondrial fission; (5) RCAN1, JNK, and Mff all bind together in HK-2 cells; (6) RCAN1 overexpression increased mitochondrial dysfunction and apoptosis, but these were alleviated after JNK silencing. To the best of our knowledge, this is the first study to describe the role of the RCAN1/JNK pathway as a mechanism responsible for AKI via the mediation of Mff-required mitochondrial fission.

RCAN1 is a multifunctional protein whose primary function is related to the regulation of calcineurin activity and mitochondrial function [21]. It has been reported that RCAN1 is significantly upregulated in neuronal cells of patients with DS and Alzheimer’s disease, and β cells in type 2 diabetes, accompanied by disruption of mitochondrial homeostasis [6, 8, 19, 22–24]. However, cardiac-specific RCAN1 overexpression in mice protects the heart from various pathological stresses, including I/R [18]. These controversial results indicate that RCAN1 functions related to mitochondrial regulation appear to be dependent on different cellular contexts.

Previous studies have suggested that mitochondrial dysfunction plays a vital role in the pathogenesis of AKI [25–27]. However, whether RCAN1 involves in mitochondrial regulation in AKI remains unknown. In this study, we found that RCAN1 was significantly upregulated in I/R- or cisplatin-AKI. To further clarify the role of RCAN1 in AKI, we crossed RCAN1 floxed allele mice with TECs-specific Cdh16-Cre mice to generate TECs-specific RCAN1 knockout mice, and found that RCAN1 knockout reduced renal injury, mitochondrial dysfunction, and apoptosis in AKI.

To further explore the molecular mechanism of mitochondrial regulation by RCAN1, we cultured HK-2 cells and constructed models using HR and cisplatin. It was found that HR and cisplatin-induced mitochondrial damage, such as mitochondrial fragmentation, decreased mitochondrial membrane potential, mitochondrial ROS overproduction, insufficient ATP supply, Cyt-c leakage, and activation of caspase-9-dependent apoptosis, was improved to some extent by RCAN1 silencing. A growing number of studies have shown that excessive mitochondrial fission is common in HR-injured cells [10, 28]. During mitochondrial fission, Drp1 translocation is critical, and its recruitment to mitochondria requires its corresponding receptors that are located on the mitochondrial outer membrane, such as Fis1, Mff, MiD49, and MiD51 [29, 30]. Our study found the most dramatic changes in Mff and phosphorylated Mff after I/R injury and RCAN1 deletion, therefore, we focused on Mff and its phosphorylation to understand how RCAN1 controls mitochondrial fission.

Many studies have found that Mff and the upstream JNK signaling pathway play an important role in myocardial ischemia-reperfusion injury [17], diabetes [31, 32], lung cancer [33], and ox-LDL-induced endothelial cell injury [34]. However, the role of the JNK/Mff signaling pathway in AKI has not been reported. In this study, we found that I/R or cisplatin injury upregulated the expression of RCAN1 and led to the phosphorylation of JNK and Mff. However, RCAN1 knockout reduced their phosphorylation. Furthermore, activation of JNK increased the expression of Mff and p-Mff in HR injury, triggering excessive mitochondrial fission. More importantly, our data identified that RCAN1 overexpression-induced apoptosis and Mff-mediated mitochondrial dysfunction were alleviated after JNK silencing. Therefore, we speculated that RCAN1 might be located upstream of the JNK/Mff signaling pathway. Surprisingly, the Co-IP results suggest that RCAN1 directly interacted with JNK and Mff in HK-2 cells. Therefore, this is the first study to describe the relationship between RCAN1 and AKI in detail. The results remind us that RCAN1 was not present in the mitochondria. We concluded that RCAN1 promoted phosphorylation of Mff by binding to downstream JNK, and promoted Drp1 migration to mitochondria, ultimately leading to excessive mitochondrial division and apoptosis.

Recent evidence shows that the activation of mitochondrial fission is related to mitophagy induction [14]. The PINK1/Parkin signaling pathway plays a central role in regulating mitophagy [35]. In addition, BNIP3 can also interact with LC3 family proteins via its LIR motifs facing the cytosol, thereby mediating mitophagy [36]. Damaged mitochondria are then labeled with ubiquitin and bind with LC3 to form mitochondrial autophagosomes [37]. We examined the role of RCAN1 in mitophagy and demonstrated that RCAN1 might mediate LC3 accumulation and autophagosome clearance, resulting in dysfunctional tubular autophagy by regulating PINK1/Parkin and BNIP3-mediated mitophagy in AKI.

In conclusion, renal I/R and cisplatin injury induced the upregulation of RCAN1, leading to increased phosphorylation of JNK. JNK activation upregulated downstream Mff and promoted Mff-mediated mitochondrial division, ultimately resulting in apoptosis of TECs. Our findings shed light on the role of RCAN1 in AKI and suggest that RCAN1 might be a new target for the treatment and prevention of AKI.

## Materials and methods

### Animals

Animal maintenance and all experiments were performed in accordance with the Chinese Ethics Community Guidelines and approved by the Center for Animal Experiment, Wuhan University. Mice were maintained in an air-conditioned room (22 ± 2 °C) under a 12 h/12 h light/dark cycle and allowed water and standard chow. TECs-specific cadherin-16 (Cdh16)-Cre transgenic mice were purchased from Shanghai Model Organisms Center, Inc (Shanghai, China). RCAN1^flox/flox^ (RCAN1^f/f^) mice were generated by CRISPR/Cas9-stimulated homologous recombination. To generate mice with RCAN1 deletion specifically in TECs, RCAN1^f/f^ mice were crossed with Cdh16-Cre mice (Fig. S1A). All mice were crossed on a C57BL/6 background for at least three generations. The genotype of RCAN1^f/f^, Cdh16-Cre + , and RCAN1-conditional knockout (RCAN1^CKO^) mice was confirmed by PCR using specific primers (Fig. S1B). Primer sequences are described in Table S1.

### Renal I/R-AKI and cisplatin-AKI models in vivo

Renal ischemia AKI was induced using I/R injury model (8–10 weeks old male mice, *n* = 6–8/group). The mice were assigned to 4 groups (RCAN1^f/f^ Sham, RCAN1^CKO^ Sham, RCAN1^f/f^ I/R-AKI, and RCAN1^CKO^ I/R-AKI). In brief, renal ischemia/reperfusion was induced by the following procedure: mice were placed in a prone position on a heated surface covered with an absorbent pad; the dorsal skin along the midline of the mouse (~1.5 cm) was cut using scissors and forceps; a small incision was made through the bilateral flank muscle and fascia above the kidney and the kidney was exteriorized; ischemia was applied to mice for 30 min by clamping the right renal pedicle and moving the nontraumatic clamps. The sham group was only treated with the sham operation, but no ischemia. Mice were sacrificed after 24 h, and blood samples and kidneys were collected.

To induce cisplatin-AKI (Cis-AKI), mice received a single intraperitoneal (i.p.) injection with cisplatin (20 mg/kg body weight). The mice were assigned to 4 groups (RCAN1^f/f^ Sham, RCAN1^CKO^ Sham, RCAN1^f/f^ Cis-AKI, and RCAN1^CKO^ Cis-AKI), and control mice were injected with 0.9% saline as described previously [38]. After 72 h cisplatin treatment, mice from all groups were sacrificed. The blood was collected for serum creatinine (Scr) and blood urea nitrogen (BUN) measurements, and the isolated serum was stored at −80 °C for further analysis. Kidney tissues for histological analysis were fixed in 4% paraformaldehyde (PFA). The remaining kidney tissues were stored at −80 °C for protein analysis.

### Serum biochemical analysis

Blood was collected and serum samples were collected by centrifugation at 1500 rpm for 10 min. The levels of Scr and BUN were evaluated using a Creatinine Assay kit (C011-1-1) and a Urea Assay Kit (C013-1-1, Jiancheng Bioengineering Institute, Nanjing, China), respectively.

### Histopathology, immunohistochemistry (IHC), immunofluorescence (IF), and transmission electron microscopy

Kidneys from mice in all groups were fixed in 4% PFA for 24 h at room temperature and embedded in paraffin. Sections were prepared for hematoxylin and eosin (HE) staining, and the tubular injury index was determined as previously described [13]. In addition, for IHC staining, the renal sections were incubated with primary antibody anti-RCAN1 (A5326, ABclonal, Wuhan, China) overnight at 4 °C and then with HRP-conjugated secondary antibody (Beijing Fir Jinqiao, Beijing, China) and the DAB substrate. Micrographs of the stained sections were captured by light microscopy (Zeiss Imager A2, Germany) and quantified using ImageJ (NIH, Bethesda, MD, USA).

Cells and frozen renal tissue sections were fixed with 4% PFA for 30 min at room temperature, washed with PBS, and permeabilized with 0.3% Triton X-100 for 10 min. After blocking in 5% BSA for 30 min, samples were immunolabeled with primary antibodies overnight at 4 °C. The primary antibodies used in the present study were as follows: NGAL (GB111134, Servicebio, Wuhan, China), RCAN1 (ab185931, Abcam, UK), RCAN1 (sc-377507, Santa Cruz Biotechnology, USA), JNK (sc-7345, Santa), Phospho-Drp1^S616^ (3455), COX IV (11967), P62 (5114), BNIP3 (44060), and p-JNK (9255) from Cell Signaling Technology (CST, MA, USA). Cells and renal tissue were then incubated with FITC- or TRITC-conjugated secondary antibody (1:200) for 1 h at 37 °C. Nuclei were visualized by staining with DAPI (G1012, Servicebio) for 10 min at room temperature. Digital images of the sections were captured using confocal microscopy (Leica TCS SP8, Germany), and the results were analyzed using Image-Pro Plus 6.0 software (Media Cybernetics, Inc., USA).

Briefly, 1 mm^3^ fresh renal cortex was placed in an electron microscopy fixative (G1102, Servicebio) at 4 °C overnight. The kidney tissues were then dehydrated in an ascending series of ethanol, and embedded in epoxy resin. Ultrathin sections (70 nm) were cut by an ultramicrotome (Leica Ultracut), stained with uranyl acetate and lead citrate, and then, examined in a transmission electron microscope (Hitachi, HT7700, Japan) at 60-80 kV.

### Terminal deoxynucleotidyl transferase dUTP nick-end labeling (TUNEL) assay and Annexin V-FITC apoptosis detection

Apoptotic cell death in kidney sections was determined using TUNEL staining (Promega, Madison, WI, USA). Briefly, kidney sections were deparaffinized and pretreated with 0.1 M sodium citrate, pH 6.0, at 65 °C for 30 min and then incubated with a TUNEL reaction mixture for 1 h at 37 °C in a dark chamber; the nuclei were labeled by DAPI. Positive staining with nuclear DNA fragmentation was detected by fluorescence microscopy (Zeiss, Germany). Each section was selected for ten representative fields randomly and the TUNEL-positive cells per mm^2^ were counted.

Flow cytometric analysis was performed using the Annexin V-FITC/PI apoptosis detection kit (abs50001, Absin, Shanghai, China) to evaluate the percentage of apoptotic cells. Cells were harvested, washed twice with cold PBS, and resuspended in 100 μL of binding buffer. Cells were stained with 5 μL of annexin V-FITC for 15 min and 5 μL of PI for 5 min at room temperature in the dark, and then measured by laser eight-color flow cytometer (FACSCalibur, BD Biosciences, San Jose, CA, USA) and quantified using FlowJo 7.6 software.

### Fractionating proteins of cytoplasm and mitochondria

Cytoplasmic and mitochondrial proteins were fractionated according to the manufacturer’s instructions (C3601, Beyotime Biotechnology, Shanghai, China). β-actin was used as the loading control for cytoplasm and COX IV for mitochondria.

### Western blotting analysis and co-immunoprecipitation

Kidney cortex and HK-2 cells were harvested and lysed with a lysis buffer (50 mM Tris (pH7.4), 150 mM NaCl, 5 mM EDTA, 1% Triton X-100, 1% Glycerol, 1 mM NaF, 1 mM β-Glycerol phosphate, 0.1 mM Na_3_VO_4_, and 60 mM Octyl β-D-glucopyranoside) containing a protease inhibitor cocktail and PMSF. Protein lysates were prepared and centrifuged at 13,000 rpm and 4 °C for 15 min to remove insoluble materials. Protein concentration was determined using a BCA protein assay kit (P0010S, Beyotime). An equivalent quantity of protein, including total protein, cytoplasmic protein, or mitochondrial protein was separated on an 8 ~ 15% SDS-PAGE gel, and transferred to nitrocellulose membranes (HATF00010, Millipore, Germany). The membranes were blocked with non-fat milk (5%) in TBST buffer for 2 h, then the nitrocellulose membranes were probed with various primary antibodies overnight at 4 °C, and then incubated with secondary antibodies conjugated to HRP at room temperature for 2 h. Next, the membranes were washed with TBST and visualized on X-ray film using enhanced chemiluminescence reagent (P0018M, Beyotime). The optical density of each target protein band was assessed using Quantity One (Bio-Rad, USA) and normalized to the density of the corresponding β-actin or COX IV bands in the same sample. Detailed information about primary antibodies is listed in Table S2.

Total protein lysates (1 mg) from each sample were used for immunoprecipitation. The samples were incubated with rabbit or mouse polyclonal IgG control antibodies, anti-RCAN1 (sc-377507, Santa), anti-JNK (sc-7345, Santa), anti-Mff (84580, CST), or anti-GFP (sc-9996, Santa). Then, the lysates were rotated overnight at 4 °C. Subsequently, a total of 40 μL resuspended volume of protein A/G magnetic beads were added into the lysates, and the mixture continued rotating for another 2 h. After washing and denaturing with immunoprecipitation buffer, the eluted proteins were immunoblotted with anti-RCAN1, anti-GFP, anti-JNK, and anti-Mff as described above.

### Cell culture, treatment, and cell viability assay

The human renal proximal TEC line HK-2 was cultured in DMEM medium (HyClone, USA) supplemented with 10% fetal bovine serum (FBS, Gibco, CA, USA), 100 U/mL penicillin, and 100 μg/mL streptomycin in a humidified atmosphere at 37 °C with 5% CO_2_. The hypoxia and reoxygenation (HR) injury model was mimicked in vitro by 6 h of hypoxia (1% O_2_, 5% CO_2_, 94% N_2_) and 6 h of reoxygenation (21% O_2_, 5% CO_2_, 74% N_2_) according previous studies [39–41]. Cells were treated with a final concentration of cisplatin at 20 μM for 24 h [19]. To suppress and activate the JNK pathway, SP600125 (10 μM, Selleck Chemicals, USA) and Anisomycin (10 μM, Selleck Chemicals) were used 2 h before treatment [42]. Cell viability was determined by a CCK-8 assay kit. Briefly, 10 μL of CCK-8 solution was added to each well containing 100 μL of the medium. After incubating for 2 h, the absorbance was determined at 450 nm.

### RNA interference and RCAN1 overexpression

Small interfering RNA (siRNA) of RCAN1 (si-RCAN1) (sense: GCUCAGACCUUACACAUAGTT; antisense: CUAUGUGUAAGGUCUGAGCTT) or JNK (si-JNK) (sense: GUUCCCAGGUACAGAUCAUTT; antisense: AUGAUCUGUACCUGGGAACTT) and negative control (si-NC) were purchased from GenePharma (Suzhou, China). The si-NC, si-RCAN1, and si-JNK were individually transfected to subconfluent HK-2 cells at a final concentration of 50 nM using Lipofectamine RNAiMAX kit (Thermo Fisher, Waltham, MA, USA) according to the manufacturer’s instructions. Transfection reactions were carried out in serum-free Opti-MEM (Thermo Fisher). After transfection for 24 h, cells were cultured in serum-containing media and prepared for different experiments.

Considering that the expression level of RCAN1.1 S but not RCAN1.1 L increased in the kidney of I/R injury mice, and in the HK-2 cells with HR and cisplatin stimulus, we overexpressed RCAN1.1 S in HK-2 cells. In brief, the pCMV-EGFP-RCAN1.1 S (GFP-RCAN1) plasmid was designed and produced by GenePharma (Suzhou, China). HK-2 cells were then transfected with GFP-RCAN1 using lipofectamine 3000 reagent (Thermo Fisher) at ~70% confluency [13].

### Mitochondrial morphology, mitochondrial membrane potential, and mitochondrial ROS

Mitochondrial morphology in live cells was observed by staining with MitoTracker Deep Red (100 nM, M22426, Thermo Fisher) followed by confocal microscopy (Leica TCS SP8). The mitochondrial length was analyzed using Image-Pro Plus 6.0 software (Media Cybernetics).

Mitochondrial membrane potential was determined using the JC-1 assay (T3168, Thermo Fisher) according to manufacturer’s protocol. Cells were washed with PBS and then stained with JC-1 probe for 30 min at 37 °C/5% CO_2_ in the dark. Subsequently, PBS was used to remove free probe, and images were captured using confocal microscopy (Leica TCS SP8). The red-to-green fluorescence ratio was employed to evaluate the changes in mitochondrial membrane potential. To determine immunofluorescence, the red/green immunosignals were converted into an average grayscale intensity and subsequently analyzed using Image-Pro Plus 6.0 software (Media Cybernetics).

MitoSOX Red mitochondrial superoxide indicator (5 μM, M36008, Thermo Fisher) was used to stain mitochondrial ROS. In brief, cells were stained with the MitoSOX Red for 30 min at 37 °C/5% CO_2_ in the dark. Samples were subsequently washed with PBS to remove free probe. ROS quantification was determined by the fluorescence intensity of mito-ROS, based on previous study [43].

### ATP measurement

ATP levels were measured using an ATP assay kit according to the manufacturer’s instructions. Briefly, the collected cells and tissues were lysed with lysis buffer and then centrifuged at 12,000 g for 10 min at 4 °C. After that, an aliquot of the supernatant plus ATP detection solution was added to a 96-well plate. Luminescence was detected using a SpectraMax M5 MultiMode microplate reader.

### Statistical analysis

All data are presented as the mean ± SD from triplicate or more experiments performed in a parallel manner unless otherwise indicated. Statistically significant differences were determined by one-way ANOVA followed by Bonferroni’s Multiple Comparison Test using GraphPad Prism 6 software. A value of *P* < 0.05 was considered significant.

## Supplementary information

Supplemental Table 1 and 2

Supplemental Figure 1

Supplemental Figure 2

Supplemental Figure 3

Supplemental Figure 4

Supplemental Figure 5

Supplemental Figure Legends

Original full length western blots

aj-checklist

## Data Availability

All of the data and material in this paper are available when requested.
