# Supplementary material for: Regulator of calcineurin 1 deletion attenuates mitochondrial dysfunction and apoptosis in acute kidney injury through JNK/Mff signaling pathway
Source: Cell Death Dis. 2022 Sep 7;13(9):774. doi: 10.1038/s41419-022-05220-x (PMC9452577; doi:10.1038/s41419-022-05220-x)
Supplement: Supplementary file 1 — Supplemental Table 1 and 2 [file 41419_2022_5220_MOESM1_ESM.docx]

**Supplementary Table 1**. Primers used to identify WT, RCAN1^f/f^, Cdh16-Cre+ and RCAN1^CKO^ mice.

| Gene name | Primer sequence |
| --- | --- |
| Cdh-16 Cre forward  Cdh-16 Cre reverse  internal positive control forward  internal positive control reverse  Loxp1 forward  Loxp1 reverse  Loxp2 forward  Loxp2 reverse | 5′-GCTGAT CTGGCTCTCCAAAG-3′  5′-AGGCAAATTTTGGTGTAGGG-3′  5′-CAAATGTTGCTTGTCTGGTG-3′  5′-GTCAGTCGAGTGCACAGTTT-3′  5′-TTGGCTAGTGGGTGGAAGTC-3′  5′-AGACAGACACACGAGCCTCA-3′  5′-AGTGTCCCCCGAATCTATCC-3′  5′-GCTGATGGGCAGTGCTGATT-3′ |

**Supplementary Table 2**. The information of all primary antibodies in Western blotting.

| Primary antibody | Company | Cat.No. | Dilution rate |
| --- | --- | --- | --- |
| RCAN1 | Sigma-Aldrich | D6694 | 1:1000 |
| NGAL | ABclonal | A2092 | 1:1000 |
| p-Mff | ABmart | TA2365 | 1:500 |
| Cle-Caspase-3  Caspase-3 | ABmart  ABclonal | T40044  A0214 | 1:500  1:500 |
| LC3 | Abcam | ab51520 | 1:1000 |
| Fis1 | Santa Cruz Biotechnology | sc-376447 | 1:700 |
| PINK1 | Santa Cruz Biotechnology | sc-517353 | 1:700 |
| Parkin | Santa Cruz Biotechnology | sc-32282 | 1:700 |
| Kim-1 | Cell Signaling Technology | 14971 | 1:1000 |
| Pro-Caspase-3 | Cell Signaling Technology | 9662 | 1:1000 |
| Pro-Caspase-9 | Cell Signaling Technology | 9508 | 1:1000 |
| Cle-Caspase-9 | Cell Signaling Technology | 9508 | 1:1000 |
| Bax | Cell Signaling Technology | 2772 | 1:1000 |
| Bcl-2 | Cell Signaling Technology | 3498 | 1:1000 |
| p-Drp1^S616^ | Cell Signaling Technology | 3455 | 1:1000 |
| Drp1 | Cell Signaling Technology | 8570 | 1:1000 |
| Mff | Cell Signaling Technology | 84580 | 1:1000 |
| Mfn1 | Cell Signaling Technology | 14739 | 1:1000 |
| Mfn2 | Cell Signaling Technology | 9482 | 1:1000 |
| Opa1 | Cell Signaling Technology | 80471 | 1:1000 |
| P62 | Cell Signaling Technology | 5114 | 1:1000 |
| Cyt-c | Cell Signaling Technology | 4280 | 1:1000 |
| COX IV | Cell Signaling Technology | 11967 | 1:1000 |
| BNIP3 | Cell Signaling Technology | 44060 | 1:1000 |
| p-JNK | Cell Signaling Technology | 4668 | 1:1000 |
| t-JNK | Cell Signaling Technology | 9252 | 1:1000 |
| β-actin | Cell Signaling Technology | 4970 | 1:1000 |

RCAN1: regulator of calcineurin 1; NGAL: neutrophil gelatinase-associated lipocalin; LC3: microtubule associated protein1light chain3; Fis1: fission protein 1; PINK1, pten induced putative kinase 1; Parkin, parkin rbr e3 ubiquitin protein ligase; Kim-1: kidney injury molecule-1; Drp1: dynamin-related protein 1; Mff: mitochondrial fission factor; Mfn1: mitofusion1; Mfn2: mitofusion2; Opa1: optic atrophy 1.
