# Supplementary figures and images for: Regulator of calcineurin 1 deletion attenuates mitochondrial dysfunction and apoptosis in acute kidney injury through JNK/Mff signaling pathway
Source: Cell Death Dis. 2022 Sep 7;13(9):774. doi: 10.1038/s41419-022-05220-x (PMC9452577; doi:10.1038/s41419-022-05220-x)

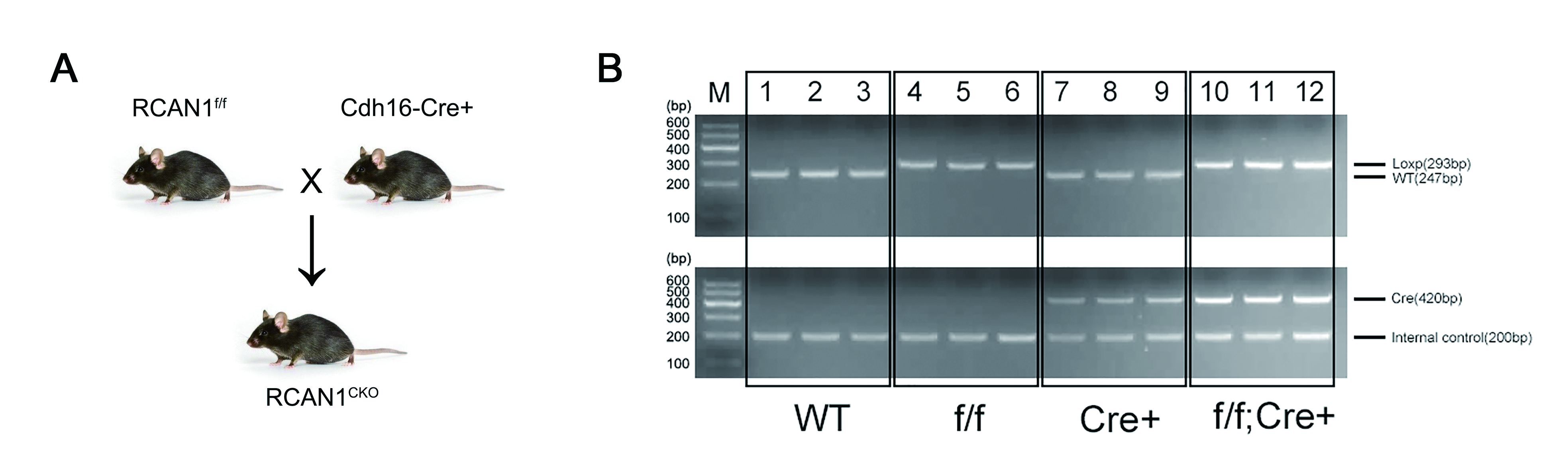

Supplement: Supplementary file 2 — Supplemental Figure 1 [file 41419_2022_5220_MOESM2_ESM.tif]

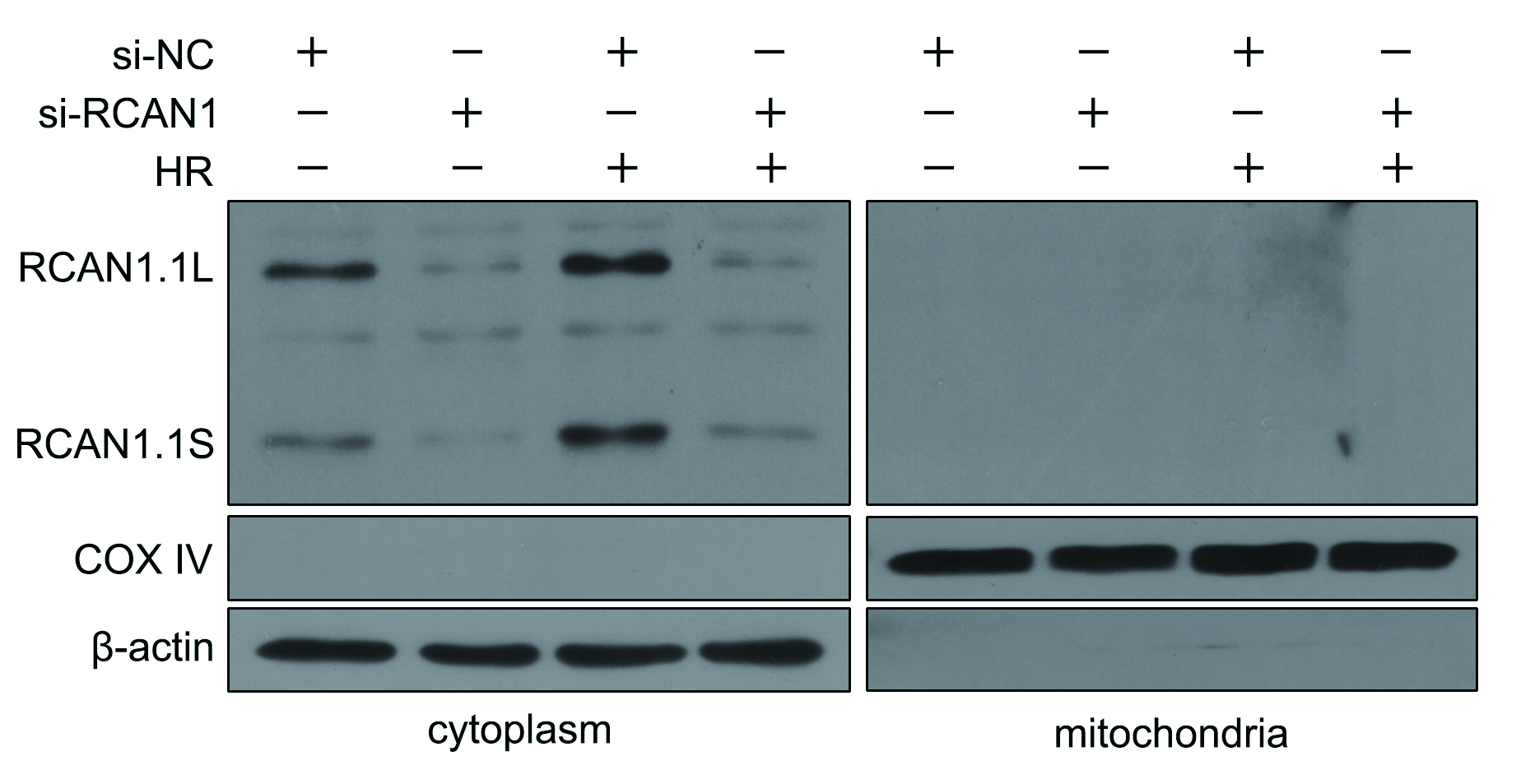

Supplement: Supplementary file 4 — Supplemental Figure 3 [file 41419_2022_5220_MOESM4_ESM.tif]

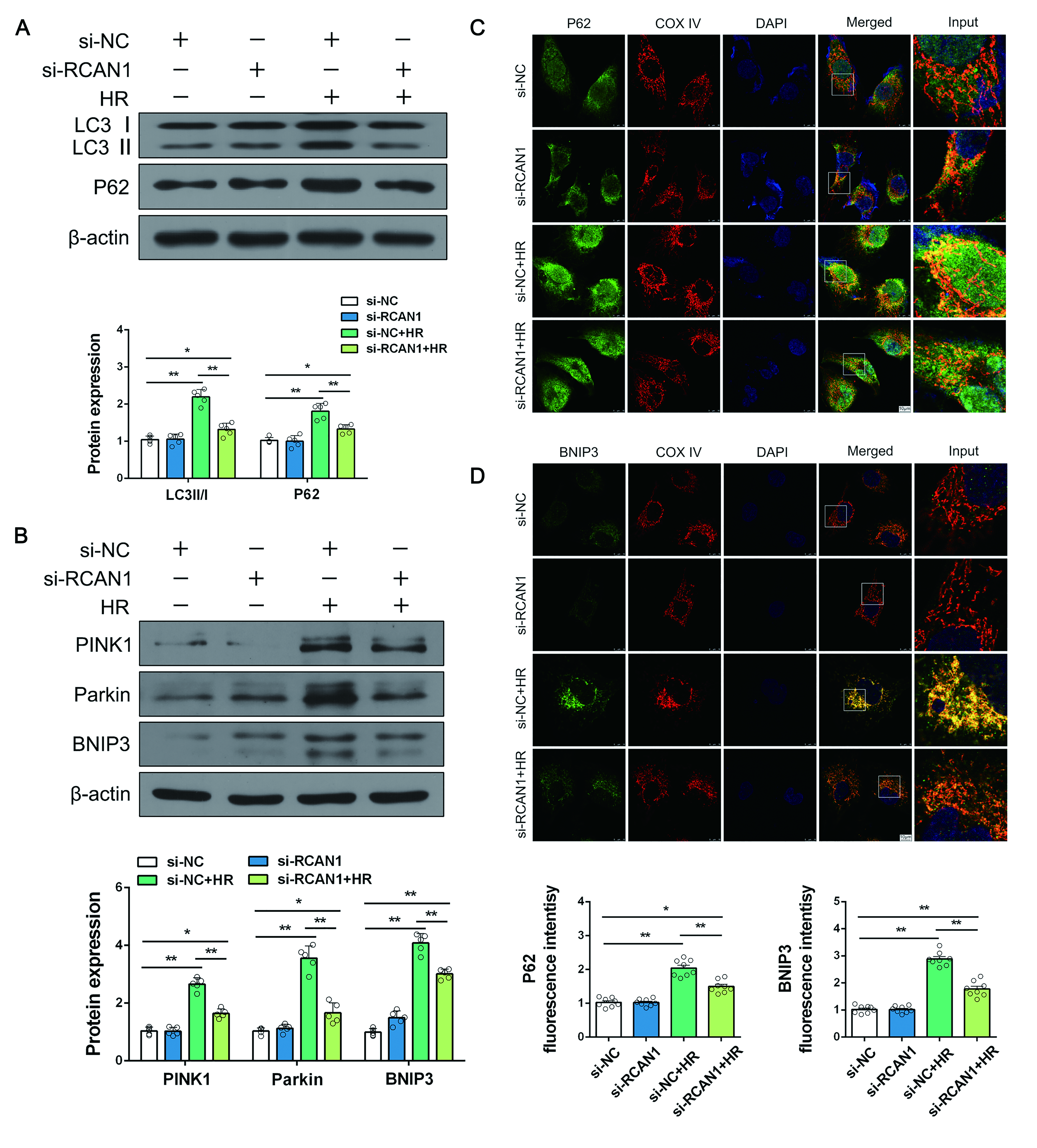

Supplement: Supplementary file 5 — Supplemental Figure 4 [file 41419_2022_5220_MOESM5_ESM.tif]

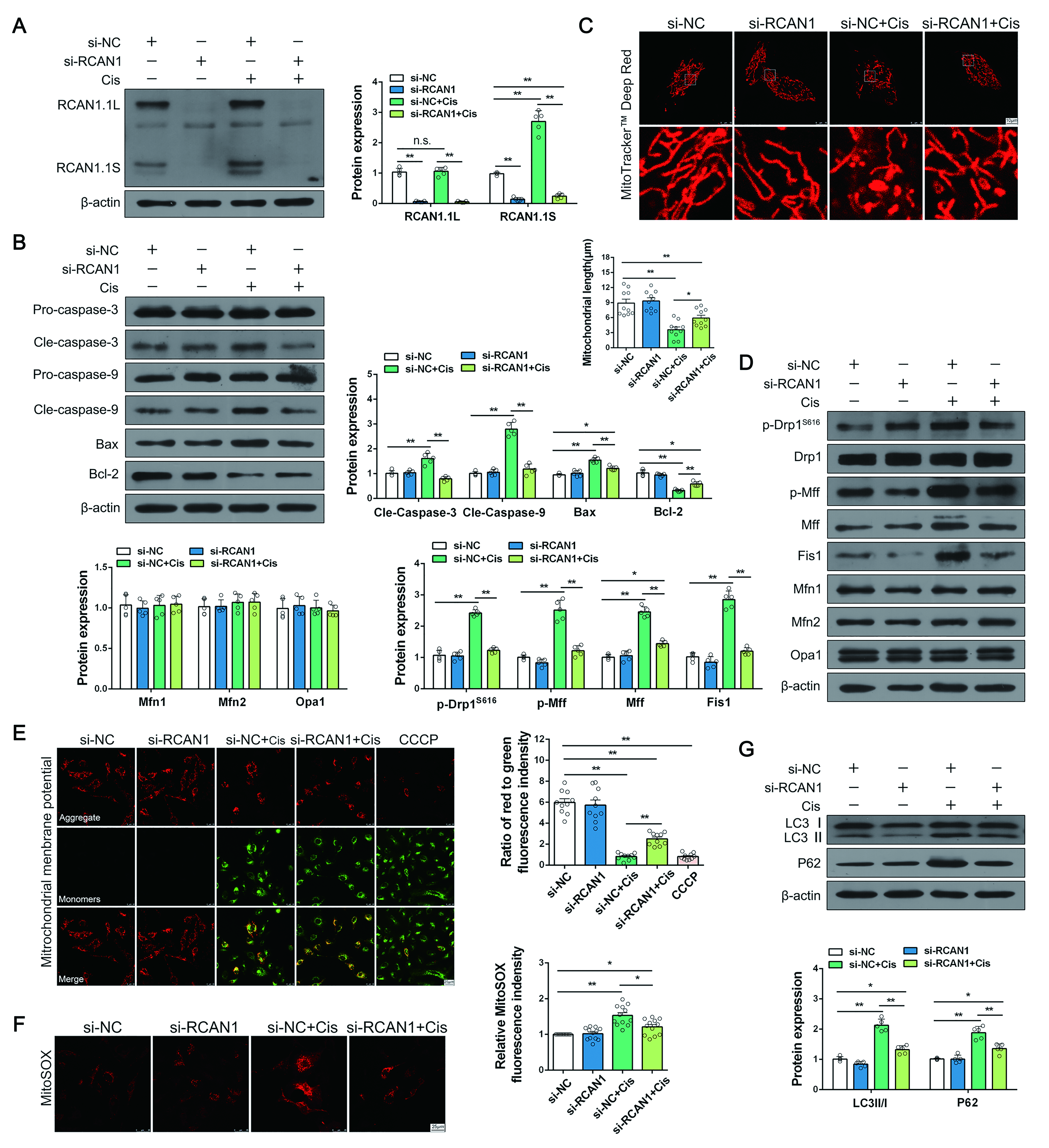

Supplement: Supplementary file 6 — Supplemental Figure 5 [file 41419_2022_5220_MOESM6_ESM.tif]
