# Supplementary material for: Regulator of calcineurin 1 deletion attenuates mitochondrial dysfunction and apoptosis in acute kidney injury through JNK/Mff signaling pathway
Source: Cell Death Dis. 2022 Sep 7;13(9):774. doi: 10.1038/s41419-022-05220-x (PMC9452577; doi:10.1038/s41419-022-05220-x)
Supplement: Supplementary file 7 — Supplemental Figure Legends [file 41419_2022_5220_MOESM7_ESM.docx]

**Supplementary figure legends**

**Fig.S1 Generation of TEC-specific RCAN1 deletion mice.** **(A)** Mating strategy to generate RCAN1 conditional knockout in mouse TECs. **(B)** Genotypes of WT, RCAN1f/f, Cdh16-Cre+， and RCAN1CKO (f/f; Cre+) were confirmed by PCR genotyping.

**Fig.S2 Deletion of tubule RCAN1 reduced renal dysfunction, mitochondrial damage, and apoptosis in Cis-AKI mice. (A)** WT mice were subjected to cisplatin injury and samples were collected on day 1, 2, and 3 after treatment. Then, the kidneys of sham operation and Cis-AKI were isolated, and the alteration of RCAN1 was monitored via Western blotting (n=6). **(B, C)** Scr and [BUN](https://www.sciencedirect.com/topics/biochemistry-genetics-and-molecular-biology/blood-urea-nitrogen) were measured using an assay kit (n=6). **(D)** [HE](https://www.sciencedirect.com/topics/biochemistry-genetics-and-molecular-biology/eosin) staining was used to observe cisplatin-mediated renal damage. Scale bar, 50 μm. n=3. **(E)** [Western blotting](https://www.sciencedirect.com/topics/biochemistry-genetics-and-molecular-biology/western-blot) was performed to analyze the expression of Pro-caspase-3, Cle-caspase-3, Bax, and Bcl-2 from sham-operated and Cis-AKI kidneys of RCAN1^f/f^ and RCAN1^CKO^ mice (n=6). **(F)** A [TUNEL](https://www.sciencedirect.com/topics/biochemistry-genetics-and-molecular-biology/transferase) assay was conducted to observe cell death. Scale bar, 50 μm. n=3. **(G)** [Western blotting](https://www.sciencedirect.com/topics/biochemistry-genetics-and-molecular-biology/western-blot) was performed to analyze the expression of p-Drp1^S616^, Drp1, p-Mff, Mff, Fis1, Mfn1, Mfn2, and Opa1 from sham-operated and Cis-AKI kidneys of RCAN1^f/f^ and RCAN1^CKO^ mice (n=6). **p* < 0.05, ***p* < 0.01.

**Fig. S3 Fractionating proteins of cytoplasm and mitochondria.** The proteins of cytoplasm and mitochondria were [fractionate](https://fanyi.so.com/#fractionate)d, and [Western blotting](https://www.sciencedirect.com/topics/biochemistry-genetics-and-molecular-biology/western-blot) was performed to analyze the expression of RCAN1, with β-actin as the loading control for cytoplasm and COX IV for mitochondria (n=3).

**Fig.S4 RCAN1 silencing alleviated HR injury through mitophagy. (A)** [Western blotting](https://www.sciencedirect.com/topics/biochemistry-genetics-and-molecular-biology/western-blot) was performed to analyze the levels of LC3 and P62 (n=5). **(B)** The expression levels of PINK1, Parkin, and BNIP3 were detected by [Western blotting](https://www.sciencedirect.com/topics/biochemistry-genetics-and-molecular-biology/western-blot) (n=5). **(C, D)** IF was used to identify the subcellular localization of P62, BNIP3, and mitochondria in HK-2 cells after HR treatment. The mitochondria were labeled with the COX IV antibody. Scale bar, 25 μm. n=3. **p* < 0.05, ***p* < 0.01.

**Fig.S5 RCAN1 silencing ameliorated cisplatin-induced mitochondrial dysfunction, autophagy, and apoptosis. (A)** HK-2 cells were treated with a final concentration of 20 μM cisplatin for 24 h, and [Western blotting](https://www.sciencedirect.com/topics/biochemistry-genetics-and-molecular-biology/western-blot) was performed to analyze the level of RCAN1 (n=5). **(B)** The expression levels of Pro-caspase-3, Cle-caspase-3, Pro-caspase-9, Cle-caspase-9, Bax, and Bcl-2 were detected by [Western blotting](https://www.sciencedirect.com/topics/biochemistry-genetics-and-molecular-biology/western-blot) (n=5). **(C)** Mitochondrial morphology of HK-2 was assessed by MitoTracker^TM^ Deep Red staining, and the average length of mitochondria was measured. Scale bar, 10 μm. n=3. **(D)** [Western blotting](https://www.sciencedirect.com/topics/biochemistry-genetics-and-molecular-biology/western-blot) was performed to analyze the expression of p-Drp1^S616^, Drp1, p-Mff, Mff, Fis1, Mfn1, Mfn2, and Opa1 in HK-2 cells (n=5). **(E)** The mitochondrial potential was observed via JC-1 staining. The ratio of red to green fluorescence was recorded to quantify the mitochondrial potential (rate). Scale bar, 25 μm. n=3. **(F)** Mitochondrial ROS levels were detected by MitoSOX and then analyzed by confocal microscopy. Scale bar, 25 μm. n=3. **(G)** [Western blotting](https://www.sciencedirect.com/topics/biochemistry-genetics-and-molecular-biology/western-blot) was performed to analyze the levels of LC3 and P62. **p* < 0.05, ***p* < 0.01.
