# Supplementary material for: Regulator of calcineurin 1 deletion attenuates mitochondrial dysfunction and apoptosis in acute kidney injury through JNK/Mff signaling pathway
Source: Cell Death Dis. 2022 Sep 7;13(9):774. doi: 10.1038/s41419-022-05220-x (PMC9452577; doi:10.1038/s41419-022-05220-x)

Fig.1

Fig.1A

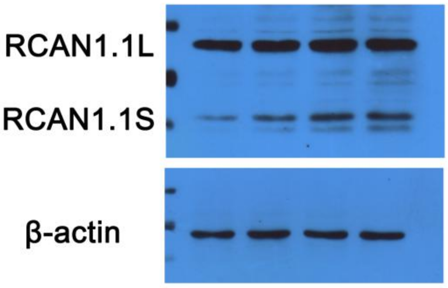

Fig.1C

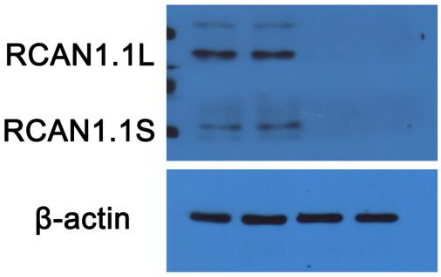

Fig.1G

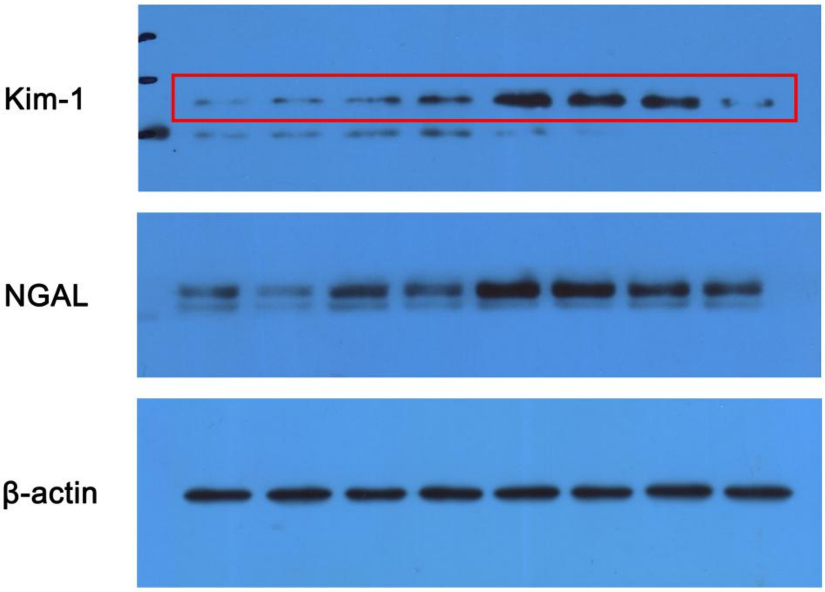

Fig.2

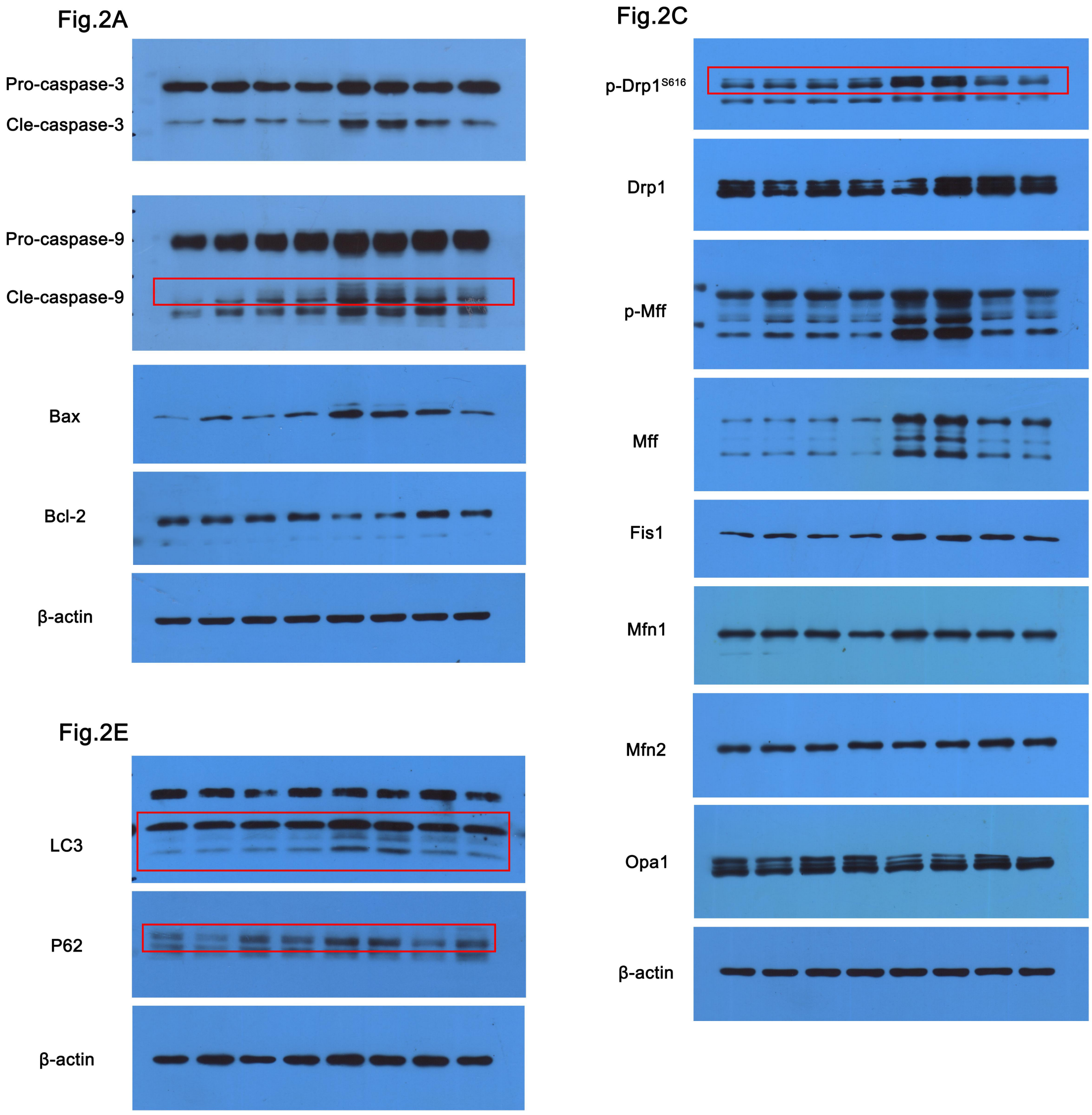
Fig.2A
Pro-caspase-3
Cle-caspase-3
Pro-caspase-9
Cle-caspase-9
Bax
Bcl-2
β-actin
Fig.2E
LC3
P62
β-actin
Fig.2C
p-Drp1S616
Drp1
p-Mff
Mff
Fis1
Mfn1
Mfn2
Opa1
β-actin

Fig.3

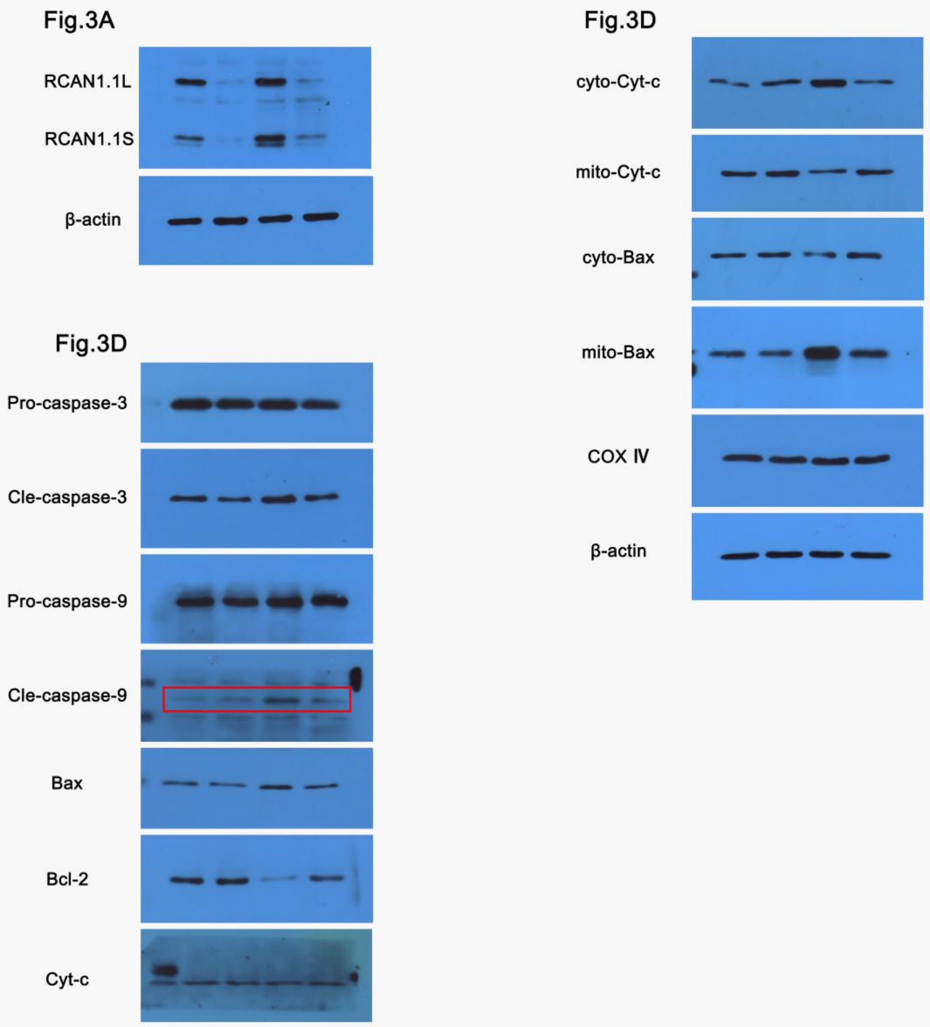
Fig.3A
RCAN1.1L
RCAN1.1S
β-actin
Fig.3D
Pro-caspase-3
Cle-caspase-3
Pro-caspase-9
Cle-caspase-9
Bax
Bcl-2
Cyt-c
Fig.3D
cyto-Cyt-c
mito-Cyt-c
cyto-Bax
mito-Bax
COX Ⅳ
β-actin

Fig.4

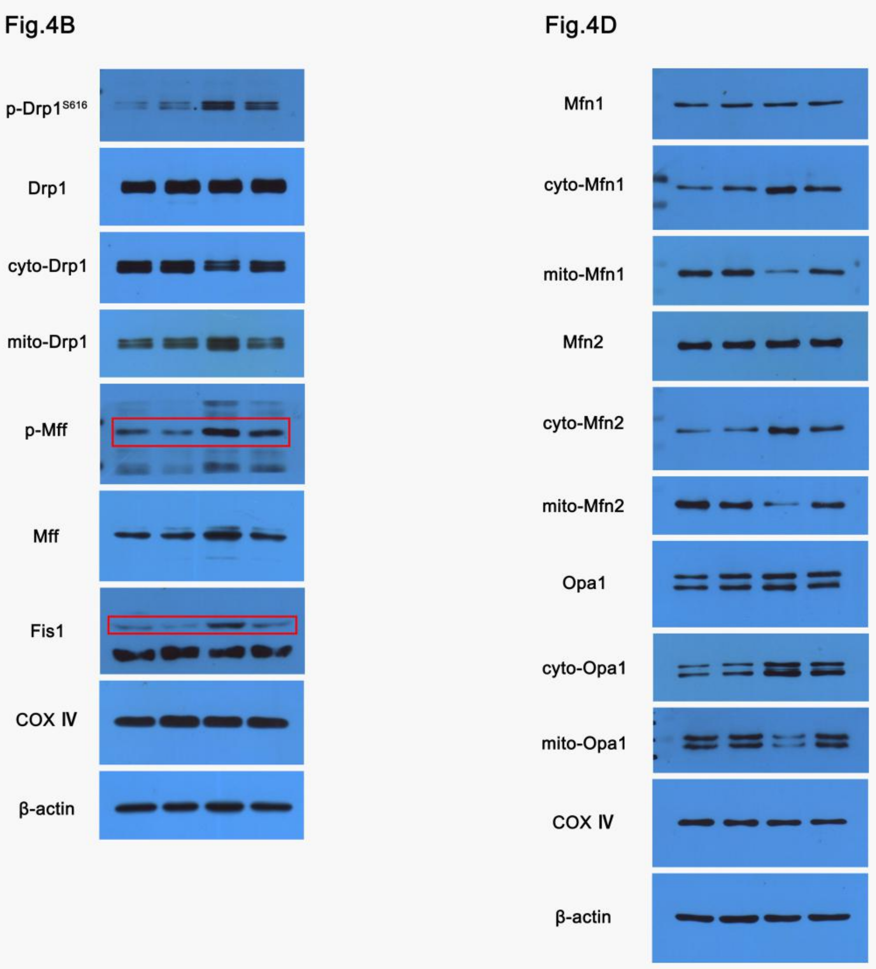
Fig.4B
p-Drp1S616
Drp1
cyto-Drp1
mito-Drp1
p-Mff
Mff
Fis1
COX IV
β-actin
Fig.4D
Mfn1
cyto-Mfn1
mito-Mfn1
Mfn2
cyto-Mfn2
mito-Mfn2
Opa1
cyto-Opa1
mito-Opa1
COX IV
β-actin

Fig.5

Fig.5A

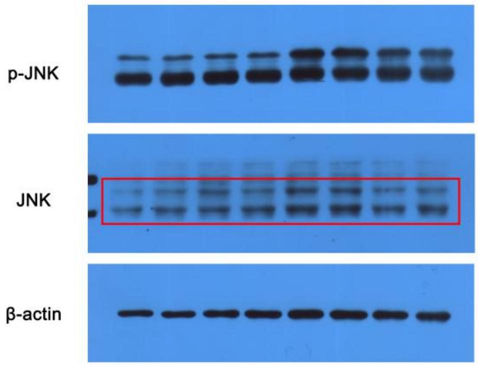

Fig.5B

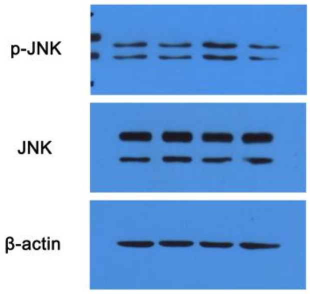

Fig.5C

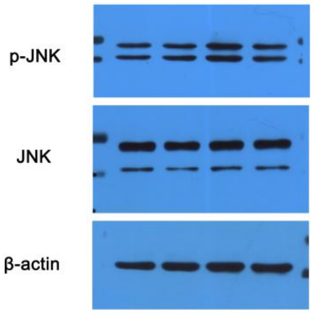

Fig.5D

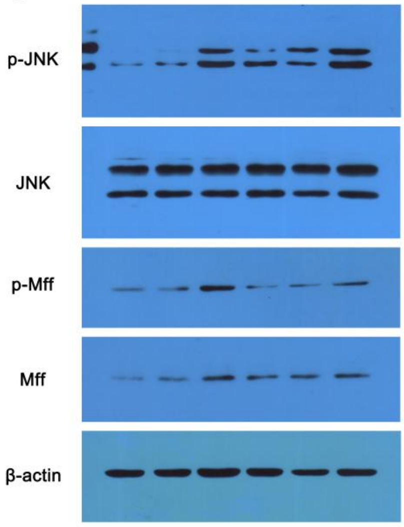

Fig.6

Fig.6A

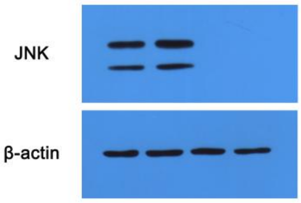

Fig.6C

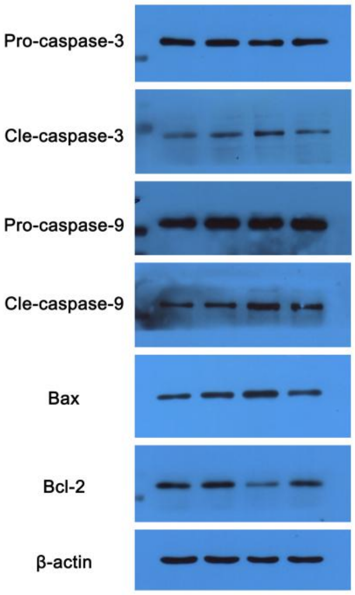

Fig.6E

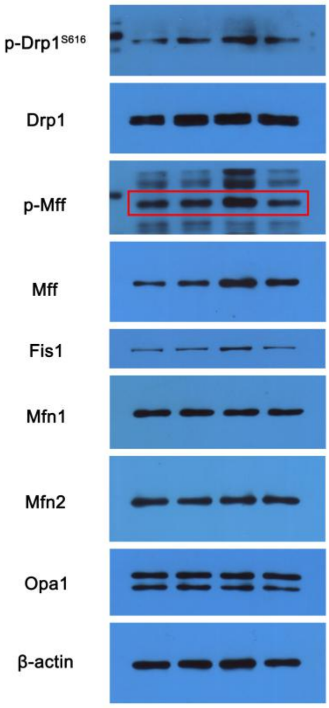

Fig.6

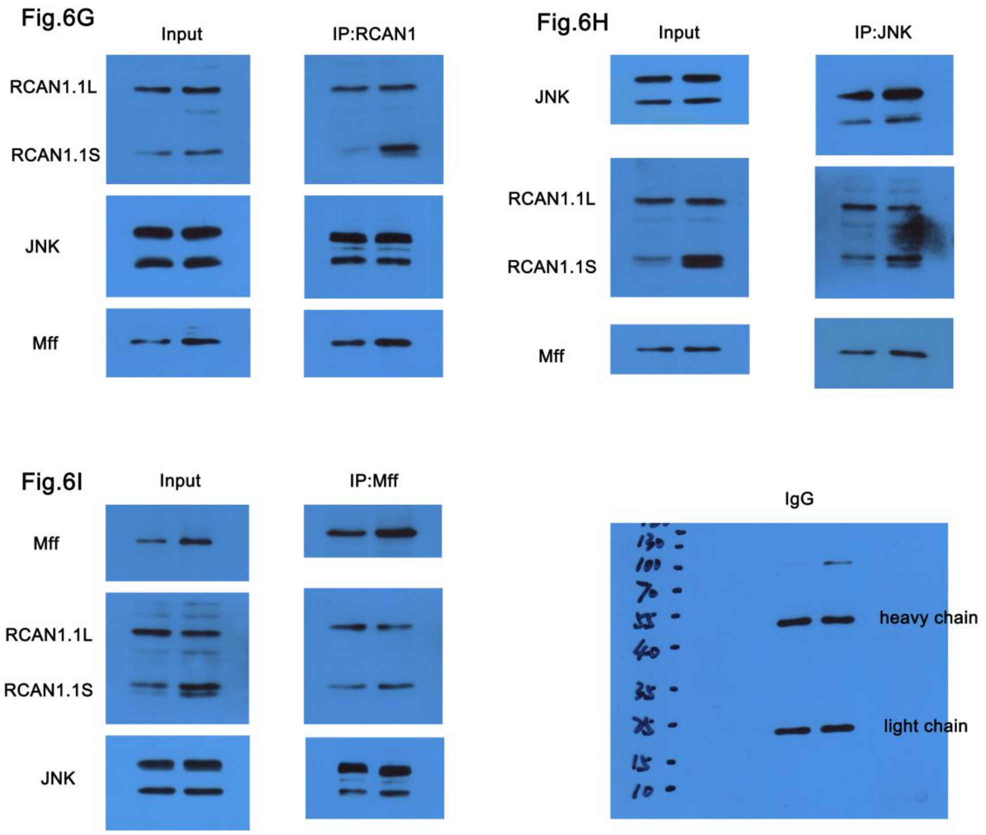

Fig.6G
Input
IP:RCAN1
RCAN1.1L
RCAN1.1S
JNK
Mff
Fig.6H
Input
IP:JNK
JNK
RCAN1.1L
RCAN1.1S
Mff
Fig.6I
Input
IP:Mff
Mff
RCAN1.1L
RCAN1.1S
JNK
IgG
heavy chain
light chain

Fig.7

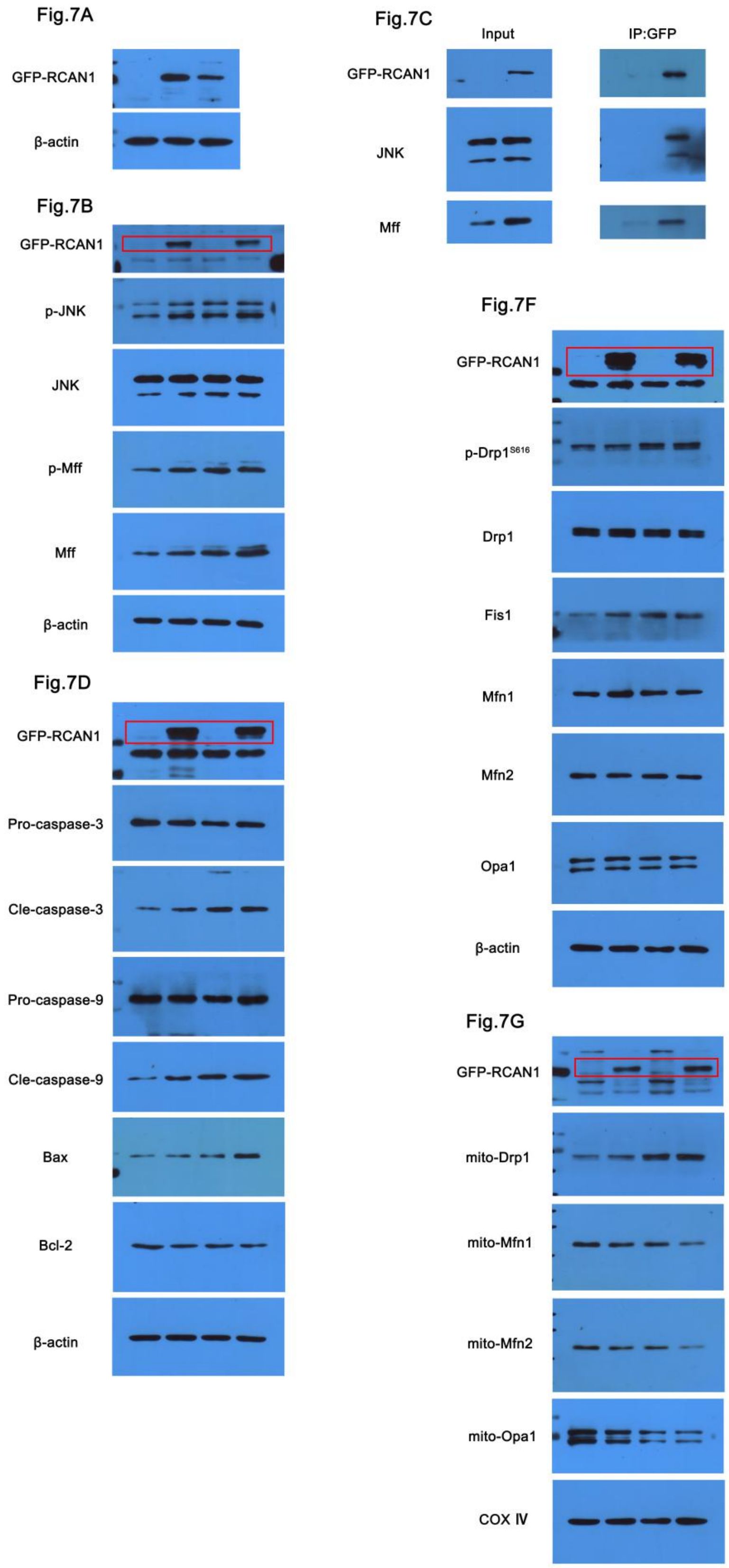

Fig.7A
GFP-RCAN1
β-actin
Fig.7B
GFP-RCAN1
p-JNK
JNK
p-Mff
Mff
β-actin
Fig.7D
GFP-RCAN1
Pro-caspase-3
Cle-caspase-3
Pro-caspase-9
Cle-caspase-9
Bax
Bcl-2
β-actin
Fig.7C
Input
IP:GFP
GFP-RCAN1
JNK
Mff
Fig.7F
GFP-RCAN1
p-Drp1S616
Drp1
Fis1
Mfn1
Mfn2
Opa1
β-actin
Fig.7G
GFP-RCAN1
mito-Drp1
mito-Mfn1
mito-Mfn2
mito-Opa1
COX Ⅳ

Fig.8

Fig.8A

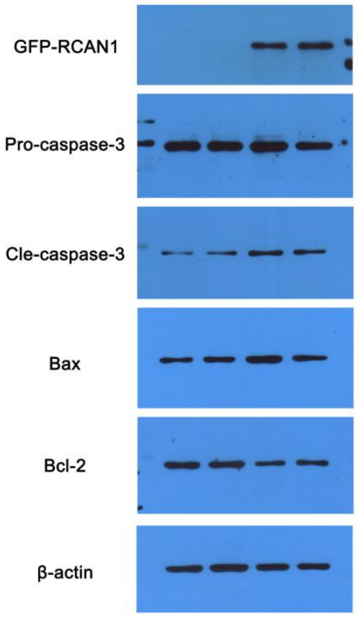

Fig.8C

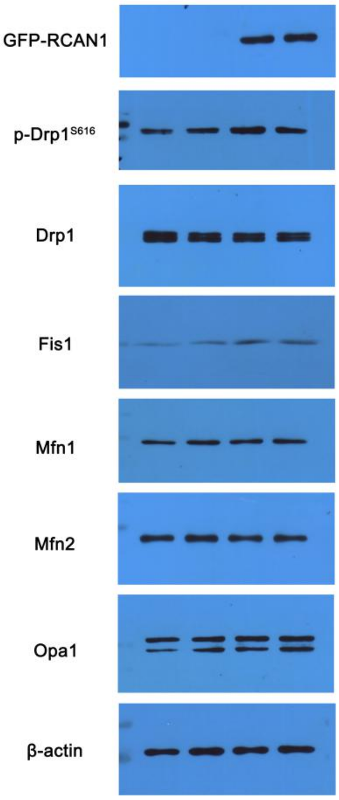

Fig.8D

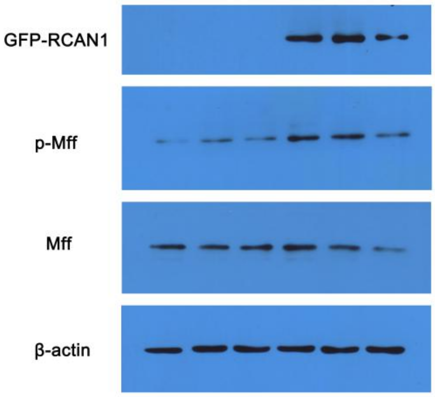

Fig.S2

Fig.S2A

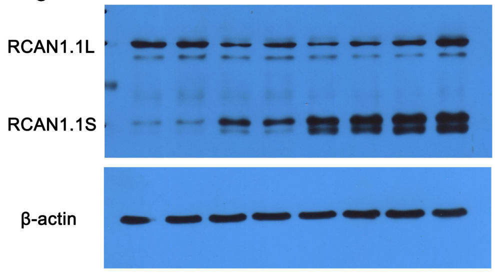

Fig.S2E

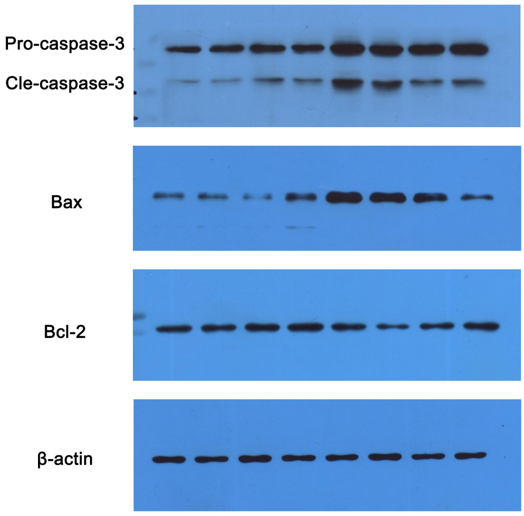

Fig.S2G

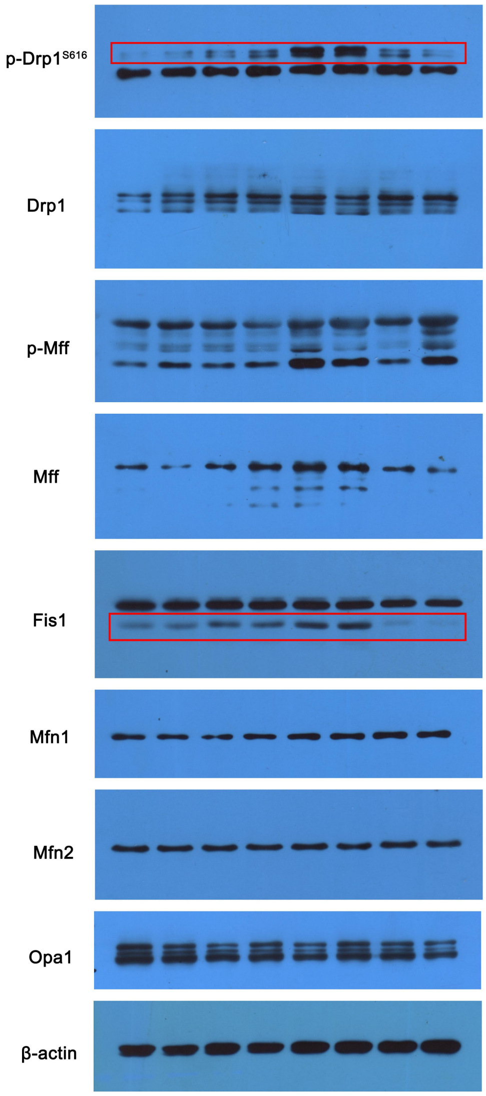

Fig.S3

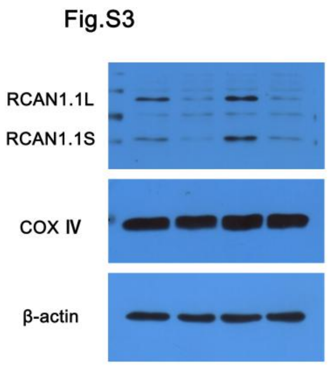

Fig.S4

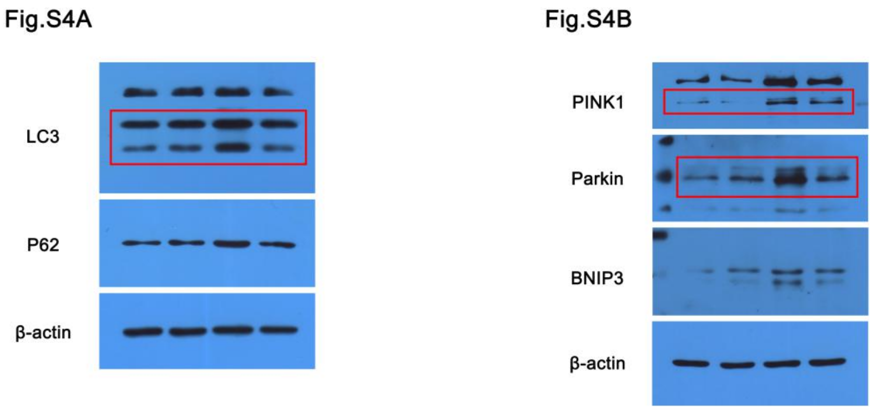

Fig.S5

Fig.S5A

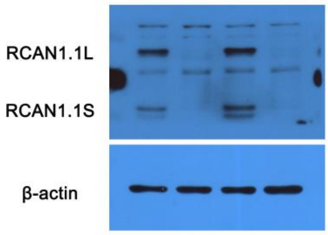

Fig.S5B

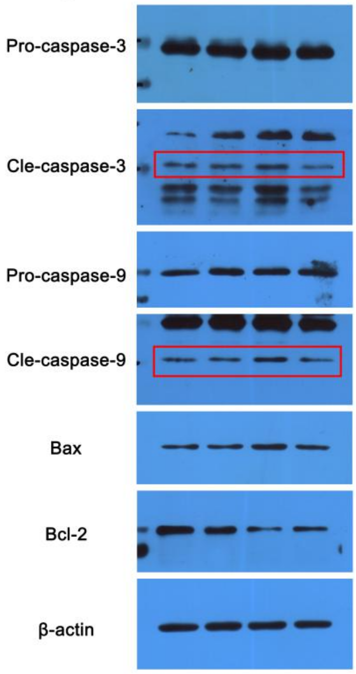

Fig.S5D

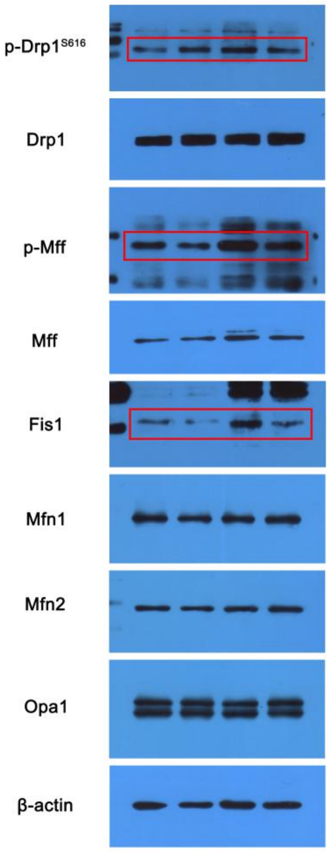

Fig.S5G

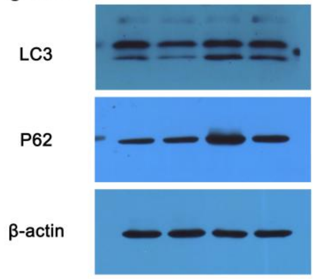

Supplement: Supplementary file 8 — Original full length western blots [file 41419_2022_5220_MOESM8_ESM.pdf]
